# 1-Deoxy-d-xylulose 5-phosphate reductoisomerase as target for anti *Toxoplasma gondii* agents: crystal structure, biochemical characterization and biological evaluation of inhibitors

**DOI:** 10.1042/BCJ20240110

**Published:** 2024-08-21

**Authors:** Flaminia Mazzone, Astrid Hoeppner, Jens Reiners, Christoph G.W. Gertzen, Violetta Applegate, Mona A. Abdullaziz, Julia Gottstein, Daniel Degrandi, Martina Wesemann, Thomas Kurz, Sander H.J. Smits, Klaus Pfeffer

**Affiliations:** 1 Institute of Medical Microbiology and Hospital Hygiene, Heinrich Heine University, Düsseldorf, Germany; 2 University Hospital Düsseldorf, Düsseldorf, Germany; 3 Center for Structural Studies, Heinrich Heine University, Düsseldorf, Germany; 4 Institute of Pharmaceutical and Medicinal Chemistry, Heinrich Heine University, Düsseldorf, Germany; 5 National Research Centre (NRC), Dokki, Cairo, Egypt; 6 Institute of Biochemistry, Heinrich Heine University, Düsseldorf, Germany

**Keywords:** anti-infective, crystal structure, DXR, DXR inhibitors, enzymatic assay, fosmidomycin, growth inhibition, parasite, SAXS, *Toxoplasma gondii*

## Abstract

*Toxoplasma gondii* is a widely distributed apicomplexan parasite causing toxoplasmosis, a critical health issue for immunocompromised individuals and for congenitally infected foetuses. Current treatment options are limited in number and associated with severe side effects. Thus, novel anti-toxoplasma agents need to be identified and developed. 1-Deoxy-d-xylulose 5-phosphate reductoisomerase (DXR) is considered the rate-limiting enzyme in the non-mevalonate pathway for the biosynthesis of the isoprenoid precursors isopentenyl pyrophosphate and dimethylallyl pyrophosphate in the parasite, and has been previously investigated for its key role as a novel drug target in some species, encompassing *Plasmodia*, *Mycobacteria* and *Escherichia coli*. In this study, we present the first crystal structure of *T. gondii* DXR (*Tg*DXR) in a tertiary complex with the inhibitor fosmidomycin and the cofactor NADPH in dimeric conformation at 2.5 Å resolution revealing the inhibitor binding mode. In addition, we biologically characterize reverse α-phenyl-β-thia and β-oxa fosmidomycin analogues and show that some derivatives are strong inhibitors of *Tg*DXR which also, in contrast with fosmidomycin, inhibit the growth of *T. gondii in vitro*. Here, ((3,4-dichlorophenyl)((2-(hydroxy(methyl)amino)-2-oxoethyl)thio)methyl)phosphonic acid was identified as the most potent anti *T. gondii* compound. These findings will enable the future design and development of more potent anti-toxoplasma DXR inhibitors.

## Introduction

*Toxoplasma gondii*, the causative agent of toxoplasmosis, is an obligate coccidian parasite member of the phylum Apicomplexa [1]. As all apicomplexans, *T. gondii* possesses a complex and heteroxenous life cycle alternating between sexual stages that occur exclusively in the intestinal epithelium of their definitive hosts (the family *Felidae*), and asexual stages that can take place virtually in any warm-blooded animal, including humans [2]. Therefore, the pathogenesis of *T. gondii* is profoundly influenced by the growth rate of its asexual stages [3]. It has reported that, in the United States, ∼11% of the population aged 6 years and older have been infected with *T. gondii* [4]. In humans, the primary routes of infection are foodborne, caused by the consumption of raw or undercooked meat that contain tissue cysts (bradyzoites), or by ingestion of contaminated vegetables or water containing sporulated oocysts [5,6]. In healthy and immunocompetent individuals, toxoplasmosis typically remains asymptomatic or manifests with flu-like symptoms, since the infection is efficiently controlled by a fully functional immune system [7,8]. On the other hand, the disease poses a significant concern in immunocompromised individuals, often leading to the reactivation of latent infection with severe clinical manifestations, such as chorioretinitis, encephalitis, pneumonitis and sepsis-like symptoms [9]. Moreover, acute *T. gondii* infections of pregnant women are a relevant risk for the unborn, as maternal-to-fetal transmission of this infection can result in devastating ophthalmic and neurological consequences as well as fatalities for the fetus [10].

Currently, the gold-standard treatment for toxoplasmosis remains the antifolate combination of pyrimethamine and sulfadiazine (PYR-SLZ) [11]. Despite the advancements in target-based drug development in the post-genomic era, human toxoplasmosis lacks sufficient treatment options [12]. Furthermore, all the current available regimens suffer from several limitations and negative aspects that compromise patient compliance and overall effectiveness. These limitations include: a lack of specificity, that can cause severe and potentially fatal side effects; and the inability to effectively target bradyzoites, the dormant cyst-form of the parasite, responsible for the latency of the infection [13]. Therefore, novel, safer and more efficient therapeutic options are urgently needed.

**Figure 1.**
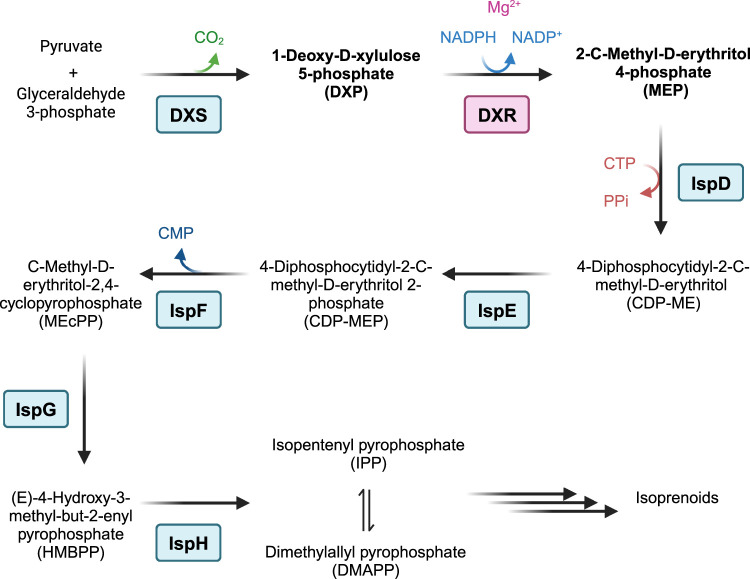
The MEP pathway for the biosynthesis of isoprenoids. Modified from Frank and Groll [86]. Created with BioRender.com.

Since its discovery, the apicoplast, a non-photosynthetic plastid organelle in the apicomplexan parasites has emerged has an attractive target for anti-infective drugs due to its absence in mammalian cells [14,15]. This plastid is responsible for essential metabolic pathways for the parasite such as fatty acid, haem, iron sulfur cluster, and synthesis of isoprenoids among others [16]. The 2-*C*-Methyl-d-erythritol 4-phosphate (MEP) pathway, also called non-mevalonate pathway, for the biosynthesis of isopentenyl pyrophosphate and dimethylallyl pyrophosphate (DMAPP), crucial metabolites for biosynthesis of isoprenoids and essential for the organism, has gathered high interest as a potential drug target (Figure 1). Its enzymes are highly conserved in plastid-bearing organisms and most gram-negative bacteria, and there are no mammalian orthologues, as humans and animals use the mevalonate pathway for the biosynthesis of isoprenoids [17–20].

**Figure 2.**
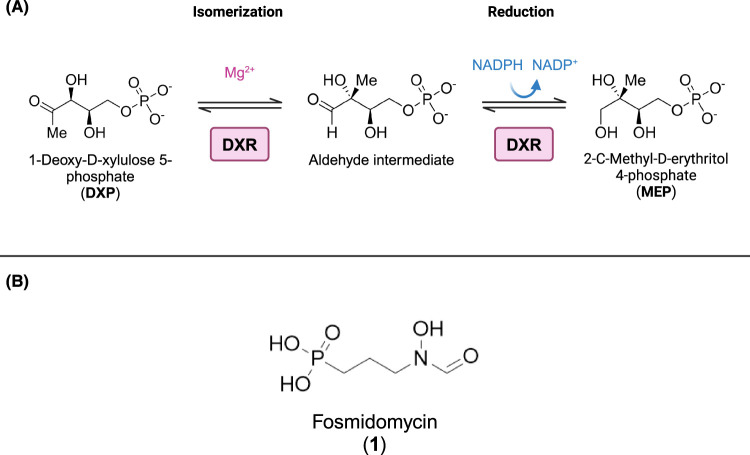
Reaction catalyzed by 1-Deoxy-d-xylulose 5-phosphate reductoisomerase (DXR) and the inhibitor fosmidomycin. (**A**) The DXR enzyme catalyzes the conversion of the substrate 1-Deoxy-d-xylulose 5-phosphate (DXP) into the product 2-*C*-Methyl-d-erythritol-4-phosphate (MEP). The catalysis occurs with dependency of a metallic dication (Mg^2+^) and NADPH as cofactors. One of the proposed mechanisms consists of a two-step reaction: first, the retro-aldol/aldol isomerization of DXP with the formation of the aldehyde intermediate [87]; then, a NADPH-depend reduction of the intermediate to MEP [88]. Modified from Kuzuyama et al. [43]. (**B**) Chemical structure of the DXR inhibitor fosmidomycin. Created with BioRender.com.

The 1-Deoxy-d-xylulose 5-phosphate reductoisomerase (DXR, IspC, EC 1.1.1.267), the second and rate-limiting enzyme in the MEP pathway, catalyzes the isomerization and the reduction of 1-Deoxy-d-xylulose 5-phosphate (DXP) into MEP. The catalysis occurs with dependency of a metallic dication (Mg^2+^) and NADPH as cofactors (Figure 2A). DXR enzymes have been extensively investigated and characterized for their key role in the pathway, and in particularly, for the development of novel antibacterial and antimalarial agents [21,22]. Moreover, several structural studies of this enzyme from different organism have been reported with high and medium resolution, such as the crystal structure of DXR from *Escherichia coli* [23], *Mycobacterium tuberculosis* [24], *Plasmodium falciparum* [25], but not from *T. gondii*.

Fosmidomycin (**1**) (Figure 2B), a natural product originally isolated from the bacteria *Streptomyces lavendulae* [26] was identified as a potent and specific inhibitor of the DXR enzymes from *E. coli* [27] and *P. falciparum* [28]. Unfortunately, this molecule faces several limitations as a clinical therapeutic related to its pharmacokinetics: short plasma half-life and rapid plasma clearance [29] as well as poor bioavailability due to the ionized nature of the phosphonate group of fosmidomycin at physiological pH [30]. Its polarity affects membrane permeability, except *E. coli* [31] that actively transports the compound. However, it is not effective in *M. tuberculosis* [32] and *T. gondii* [33] probably due to the lack of uptake systems.

Given the significance of DXR inhibitors and with the aim to identify improved inhibitors of *Tg*DXR, in the present study we successfully cloned, expressed, purified and biochemically characterized the recombinant *Tg*DXR. We report the X-ray structure of *Tg*DXR in a tertiary complex with the cofactor NADPH and the inhibitor fosmidomycin, further defined by small-angle X-ray scattering (SAXS) analysis. Moreover, we assessed the *in vitro* activity of previously described β-thia and β-oxa isosters of reverse hydroxamic acid analogues of fosmidomycin [34–36] against the activity of the recombinant *Tg*DXR enzyme and *T. gondii* proliferation. These findings could support the future design and development of novel anti-toxoplasma agents.

## Results

### Biochemical characterization

#### *Tg*DXR enzyme properties

**Figure 3.**
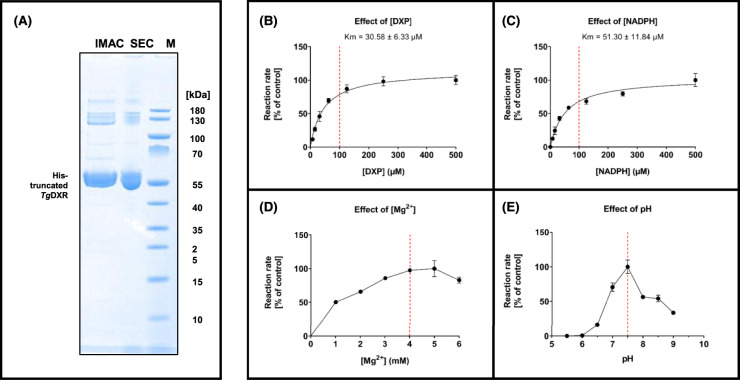
Purification of the His_10_-truncated *Tg*DXR and its optimization and kinetic characterization. (**A**) SDS–PAGE of samples taken during the purification of truncated His_10_-*Tg*DXR after immobilized metal ion affinity chromatography (IMAC) and size exclusion chromatography (SEC). Marker (M): PageRuler™ Prestained Protein Ladder, Thermo Scientific™, Thermo Fisher Scientific, Waltham, MA, U.S.A., #26616, molecular mass in kDa are indicated. Running Buffer: 20× NuPAGE™ MOPS SDS Running buffer, Thermo Fisher Scientific, Waltham, MA, U.S.A., #NP000102. (**B**) Effects of [DXP] on *Tg*DXR catalyzed reaction. *K_m_* values of DXP and NADPH are shown. (**C**) Effects of [NADPH] on the *Tg*DXR catalyzed reaction. (**D**) Effects of [Mg^2+^] on the *Tg*DXR catalyzed reaction. (**E**) Effects of pH on the *Tg*DXR catalyzed reaction. See Materials and methods section for the experimental conditions. Optimal and selected concentrations or values are indicated (red line). Data shown are means of four independent experiments, each performed in duplicate (*n* = 8) ± S.D.

We set out to functionally and structurally characterize the His_10_-*Tg*DXR, which contains the catalytic domain. To achieve this, we constructed a plasmid, which contains the catalytic centre consisting of the NADPH binding site as well as the substrate-binding site of the *Tg*DXR protein (amino acid 182–632) (Supplementary Figure S1). After expression, *Tg*DXR was purified to homogeneity as observed by a single symmetric peak on the size exclusion chromatography (SEC) profile and SDS–PAGE analysis (Figure 3). The purified *Tg*DXR was then evaluated for its enzymatic activity through a spectrophotometric assay measuring the oxidation of NADPH over time under various conditions. The optimum of its catalytic capacity was reached with 100 µM of DXP, 100 µM of NADPH, 4 mM of MgCl_2_ and a pH of 7.5 at concentration of 100 nM of *Tg*DXR (Figure 3). The kinetic parameters of *Tg*DXR were also evaluated: the *K_m_* value for DXP was determined to be 30.58 ± 6.33 µM, which is comparable with the *Tg*DXR *K_m_* value obtained previously [37] and the reported DXR *K_m_* values from other species, such as *Pf*DXR (67 µM) [38] and *Mt*DXR (47 µM) [39]; the *K_m_* for NADPH was determined as 51.30 ± 11.84 µM (Figure 3). The *V*_max_ was shown to be 0.83 mmol/min/mg, comparable to *Pf*DXR (1.04 mmol/min/mg) [38].

#### Structural characterization of the *Tg*DXR catalytic domain

*Tg*DXR was crystallised in the presence of the known inhibitor fosmidomycin using the sitting drop method. The 3 µl drops consisted of 1.5 µl protein (7.5 mg/ml in 20 mM TRIS pH 7.5, 150 mM NaCl, 2% glycerol supplemented with 1 mM fosmidomycin, 4 mM MgCl_2_ and 4 mM NADPH) mixed with crystallization buffer 1.5 µl 200 mM Na citrate tribasic, 27% PEG smear low, 150 mM HEPES pH 7.8. Crystallization was performed at 12°C and crystals appeared within a few days and grew to their final dimensions within two weeks. Before the crystals were flash-cooled in liquid nitrogen, the crystallization drops were overlaid with mineral oil and the crystals were dragged through it during crystal harvesting. A high-resolution dataset was collected and phased using the model of the DXR protein from *E. coli* (PDB entry: 1K5H) [40] revealing a dimer in the asymmetric unit.

#### The overall structure

The structure of *Tg*DXR was solved and refined to 2.5 Å resolution (data-collection and refinement statistics are summarized in Supplementary Table S2). The asymmetric unit contains one homodimer. The r.m.s. deviation between Cα atom positions within in the two subunits when they are superimposed is 0.3 Å using all 347 Cα atoms. Residues 25–472 could be modelled for both molecules from the electron-density map except the loop ranging from amino acid 182–221 and were therefore not modelled in either of the two subunits. First, we describe the overall structure for monomer A.

**Figure 4.**
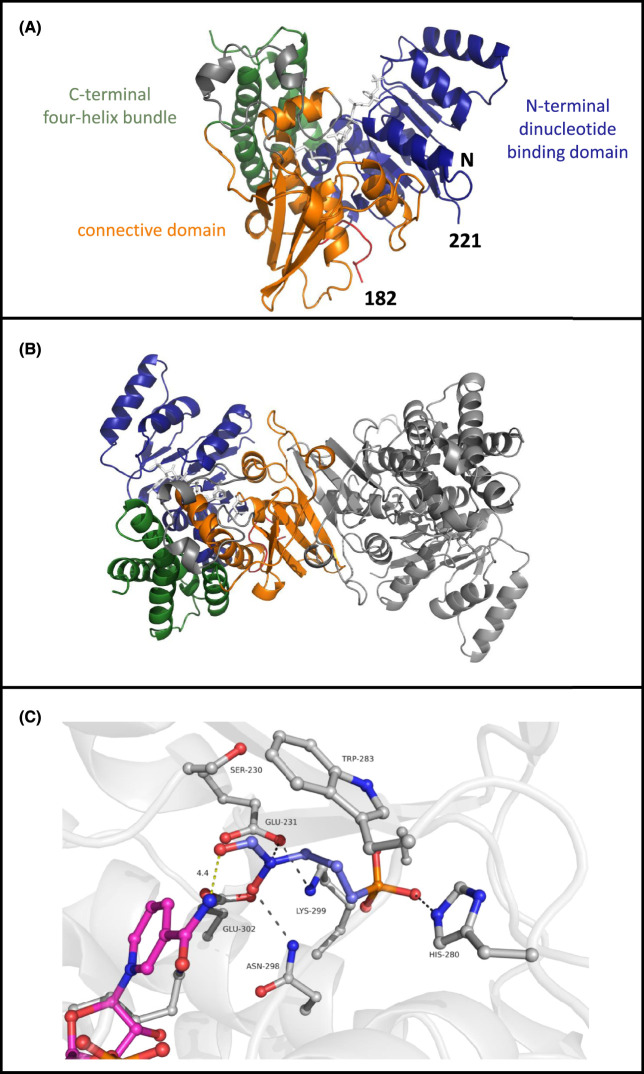
Structure of *Tg*DXR A. (**A**) Overall structure of *Tg*DXR as observed in the crystal structure. Shown is chain A which is similar to chain B, as calculated by comparing the RMSD of both monomers after superimposing which is 0.3 Å overall. Three different domains are observed and colour coded being the N-terminal nucleotide binding domain (blue) the connective domain (orange) and the C-terminal four helix bundle (green). (**B**) The dimer of *Tg*DXR is highlighted as observed in the asymmetric unit of the crystal. The dimer interface is mediated by the connective domain highlighted in orange in chain A. (**C**) The binding site of fosmidomycin is shown with in ball and stick representation. Fosmidomycin (blue) is bound via interaction with the sidechains of Glu231, His280, and Asn298. All distances are indicated with black line with a maximum distance of 3.6 Å. The distance of fosmidomycin to NADPH (purple) is 4.4 Å, which is too large to allow activity.

*Tg*DXR is composed of three domains: an N-terminal NADPH-binding domain, a connective domain and a C-terminal α-helical domain (Figure 4A). These are arranged in a V shape, where the N-terminal and C-terminal domains form the two arms and the central domain lies at the vertex.

The N-terminal domain (residues 25–171) consists of a Rossmann fold, with a β-sheet containing seven parallel β-strands (β1–β7, including a kink at residue 56 in β2), which is flanked by a total of six α-helices (α1–α6). The N-terminal domain is connected via a long loop (residues 172–227) to the catalytic domain. Of this loop, the largest part is not visible in the electron density and appears to be flexible although NADPH and the inhibitor fosmidomycin are present. The catalytic domain (residues 228–385) includes a four-stranded β-sheet ordered β9–β8–β10–β11, where β10 is positioned antiparallel to the other strands. This sheet adheres to the other two domains by virtue of a layer of helices. β8 extents into a flexible loop consisting of a broken helix α7 which then returns into the four-stranded β-sheet via α helix α8.

The C-terminal domain residues (386–472) feature a four-helix bundle. The dimer interface is created by interactions between the catalytic and connecting regions of each subunit. A twisted eight-stranded β-sheet is formed using the four β-strands of each catalytic domain, with the respective β11 strands positioned antiparallel at its central point (Figure 4B). Further antiparallel interactions between the β12 strands of each subunit are found on the concave surface of this larger sheet; interactions at the C-terminal end of the β12 strand links them to the sheet, thus forming an imperfect ten-stranded β-barrel as the core of the dimer interface.

In the electron density, NADPH could be unambiguously identified and modelled. NADPH is bound to the Rossmann fold by interactions typically observed, while the pyrophosphate moiety interacts with the consensus sequence GGGNGA, establishing further interactions with the co-substrate NADPH which is bound at the identical position as found in other DXR proteins [23,25,41,42]. The binding of the adenine and pentose phosphate moieties of NADPH is identical to that observed in the structure of the *E. coli* DXR, in contrast, the nicotinamide ring of NADPH is ordered in this fosmidomycin complex (Figure 4C).

#### Fosmidomycin binding site

Within the solvent-shielded cavity that is formed upon closure of the ‘lid’ residues 281–285 the inhibitor backbone lies parallel to the β-indole of Trp283 at a distance of ∼4 Å. The hydroxamic acid moiety of the inhibitor binds to the side-chain of Glu231. The sidechain of Glu231 itself is stabilized and positioned via interaction with Lys299 (Figure 4C). Fosmidomycin further interacts with the sidechains of Asn298 and His280. The sidechain of Glu302 also points towards the fosmidomycin, however the distance is roughly 4 Å indicating a very weak interaction or maybe this interaction is water mediated which cannot be conclusively modelled at this resolution.

#### SAXS of *Tg*DXR

**Figure 5.**
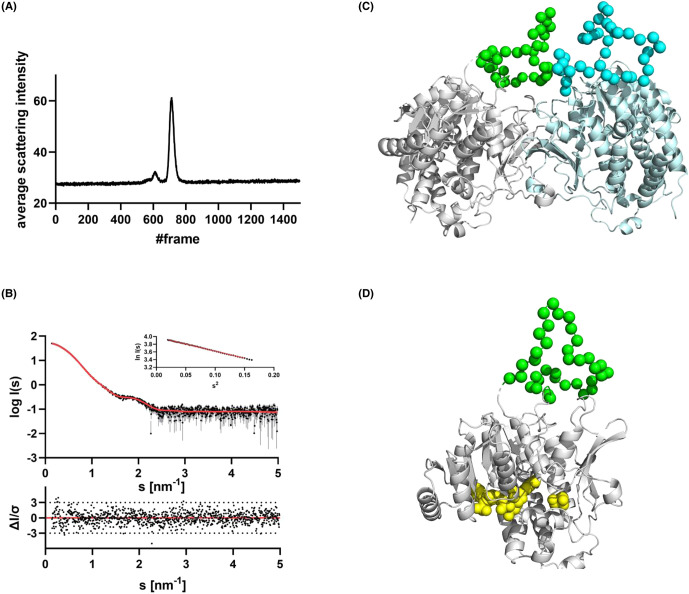
Small-angle X-ray scattering data from *Tg*DXR and the most representative (62%) EOM model. (**A**) Chromixs SEC SAXS elution profile. Each frame corresponds to 2 s. (**B**) Scattering data of *Tg*DXR. Experimental data are shown in black dots, with grey error bars. The EOM ensemble model fit is shown as red line and below the residual plot of the data is given. The Guinier plot of *Tg*DXR is added in the right corner. (**C**) The rigid body protomers of *Tg*DXR from the crystal are shown in grey and cyan cartoon representation. The determined flexible loop is shown as green and blue spheres. (The other EOM models are part of the Supplementary Information). (**D**) The SAXS completed model of *Tg*DXR is shown as monomer with the flexible loop shown in green spheres. The NADPH and fosmidomycin binding site is highlighted in yellow. It is clear that the flexible loop is remote of the active site and likely serves a more stabilizing role in the dimer of *Tg*DXR.

The *Tg*DXR protein was successfully crystallised and forms a dimeric conformation in solution. However, parts of the protein were not visible in the electron density, likely due to the flexibility, e.g. parts from the N-terminus and more importantly the flexible loop region from the *Tg*DXR protein. These loop regions are special in this *Tg*DXR variant and are not present in homologous structures. We used SAXS to determine the structure of the *Tg*DXR protein in solution. In the SEC-SAXS elution profile (Figure 5A; and Supplementary Figure S2A), *Tg*DXR elutes in one homogenous peak. Evaluation of the data revealed a dimer in solution with the same shape and orientation as determined by X-ray crystallography, with an *R_g_* value of 3.33 nm and a *D*_max_ value of 10.44 nm (Supplementary Table S3). With the Ensemble Optimisation Method (EOM), we modelled the missing amino acids from the loop region and the N-terminus to each protomer, which completed the structure. The corresponding scattering data with the EOM fit (*χ*^2^: 1.207) is shown in Figure 5B and the most representative EOM model in Figure 5C. The remodelled loops (37 amino acids each), cover the area between both protomers of *Tg*DXR. Its flexibility, evidenced by the absence of electron density in the crystal structure, led us to analyse the loop's position with EOM, resulting in a 62% occupancy in this conformation.

The loop is located at the back side of the *Tg*DXR protein (Figure 5D) and therefore does not play an immediate role in catalysis.

### *In vitro* biological evaluation

#### Evaluation of the activity of the *Tg*DXR mutants Glu321Ala, His280Ala and Asn298Ala

**Figure 6.**
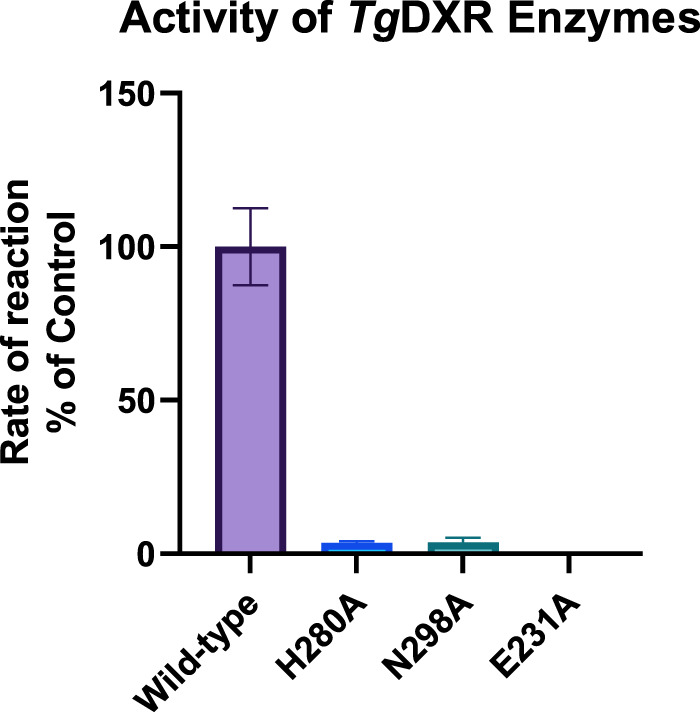
Comparison of the activities of *Tg*DXR wild-type and *Tg*DXR mutants Glu321Ala, His280Ala and Asn298Ala. Reaction rates of *Tg*DXR **wild-type** (**violet**) and *Tg*DXR mutants **His280Ala** (**H280A**, **blue**) and **Asn298Ala** (**N298A**, **green**). **Glu321Ala** (**E321A**) could not be determined. Data shown are from the means of three independent experiments each performed in duplicate (*n* = 6) ± S.D.

To identify the binding site essential for the inhibitory activity of fosmidomycin, we obtained point-mutated *Tg*DXR proteins (section ‘Site-directed mutagenesis and expression of *Tg*DXR mutants’) and conducted *in vitro Tg*DXR enzyme inhibition assays (section ‘Enzymatic assays of *Tg*DXR mutants Glu321Ala, His280Ala and Asn298Ala’). We compared the reaction rates of *Tg*DXR wild-type with *Tg*DXR mutants Glu231Ala, His280Ala, and Asn298Ala. Unfortunately, the Glu231Ala mutant could not be expressed and therefore the analysis of this mutation was not possible. Both other mutant proteins (His280Ala and Asn298Ala) displayed significantly decreased activities of only 5–6% activity compared with the wild-type DXR protein (Figure 6), indicating that these amino acids are essential for binding of the substrate DXP. This is in line with the DXR protein from *E. coli,* where mutations of the same positions and amino acids also resulted in an almost 19-fold reduction of the activity as observed for the wildtype protein [43]. Due to the low activity, the observed inhibition with fosmidomycin was measured but due to the initial low activity a valid IC_50_ could not be determined for these mutants.

#### Reverse fosmidomycin thia analogues are potent *Tg*DXR inhibitors

**Figure 7.**
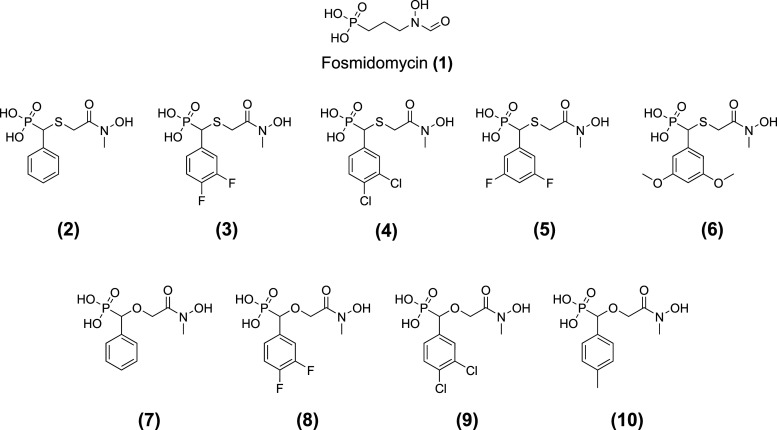
Chemical structures of DXR inhibitors investigated in this study.

Based on previous studies, demonstrating the effectiveness of reversed hydroxamic acid analogues of fosmidomycin (**1**) as DXR inhibitors, with either a bivalent sulfur or oxygen atom replacing the β-methylene group of the main chain of the linker [34–36], we tested 5 reverse thia (Figure 7, **2–6**) and 4 reverse oxa analogues (Figure 7, **7–10)** for their enzymatic activity against the purified recombinant truncated His_10_-*Tg*DXR and determined their inhibitory concentrations (IC_50_) and inhibitory constant (*K_i_*) values.

**Figure 8.**
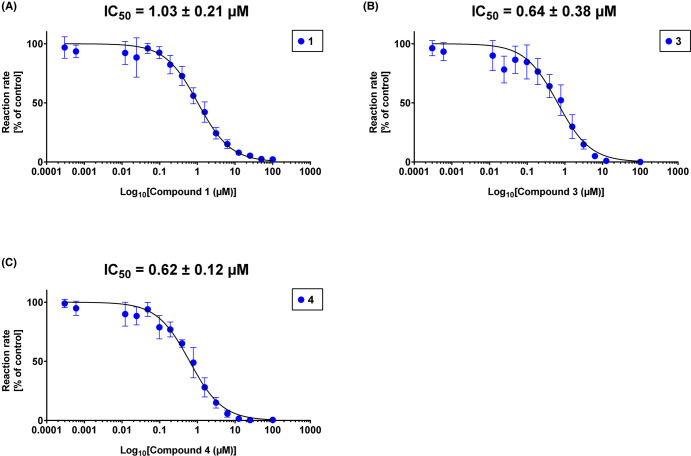
*In vitro* inhibition of *Tg*DXR by most potent DXR inhibitors. The enzymatic inhibitory activity of 1 (**A**), 3 (**B**) and 4 (**C**) were determined by enzymatic assays *in vitro*. Assays were performed in 96 well plates at 30°C, containing 100 nM of purified *Tg*DXR protein in dimeric state, 100 µM of NADPH and 4 mM of MgCl_2_ as cofactors, 100 µM of DXP as substrate in 50 mM HEPES buffer (pH 7.5) containing 50 µg/ml of bovine serum albumin (BSA). The investigated compounds were tested in a concentration range of 3.05 nM to 100 µM. Data shown are from the means of three independent experiments each performed in duplicate (*n* = 6) ± S.D. IC_50_ values of each compound are shown.

Interestingly, all the investigated compounds possessed *Tg*DXR inhibitory activity. In particular, the reverse thia isosters revealed a marked increase on the inhibitory activity compared with **1** and the cognate reverse oxa compounds (Figure 8, Table 1 and Supplementary Figure S5). Moreover, the 3,4-difluorophenyl- (**3**) and 3,4-diclorophenyl- (**4**) substituted thia analogues were the most active inhibitors with comparable submicromolar IC_50_ and *K_i_* values (Figure 8, Table 1 and Supplementary Figure S5).

**Table 1.** *In vitro* activity (IC_50_ and *K_i_* values) of DXR inhibitors against *Tg*DXR enzyme.

Compound	IC_50_ ± S.D. (µM)	*K_i_* (µM)
**1**	1.03 ± 0.21	0.31^a^
**2**	1.23 ± 0.12	0.37
**3**	0.64 ± 0.38	0.19
**4**	0.62 ± 0.12	0.19
**5**	2.45 ± 0.54	0.74
**6**	3.90 ± 1.06	1.18
**7**	5.82 ± 1.52	2.69
**8**	52.14 ± 0.34	11.00
**9**	2.44 ± 0.43	0.74
**10**	7.68 ± 0.87	2.51

a The *K_i_* value reported in previous studies is 0.09 µM [37]. Values shown in the table represent the means of three independent experiments each done in duplicate (*n* = 6) ± S.D.

#### Reverse fosmidomycin analogues inhibit the growth of *T. gondii in vitro*

**Figure 9.**
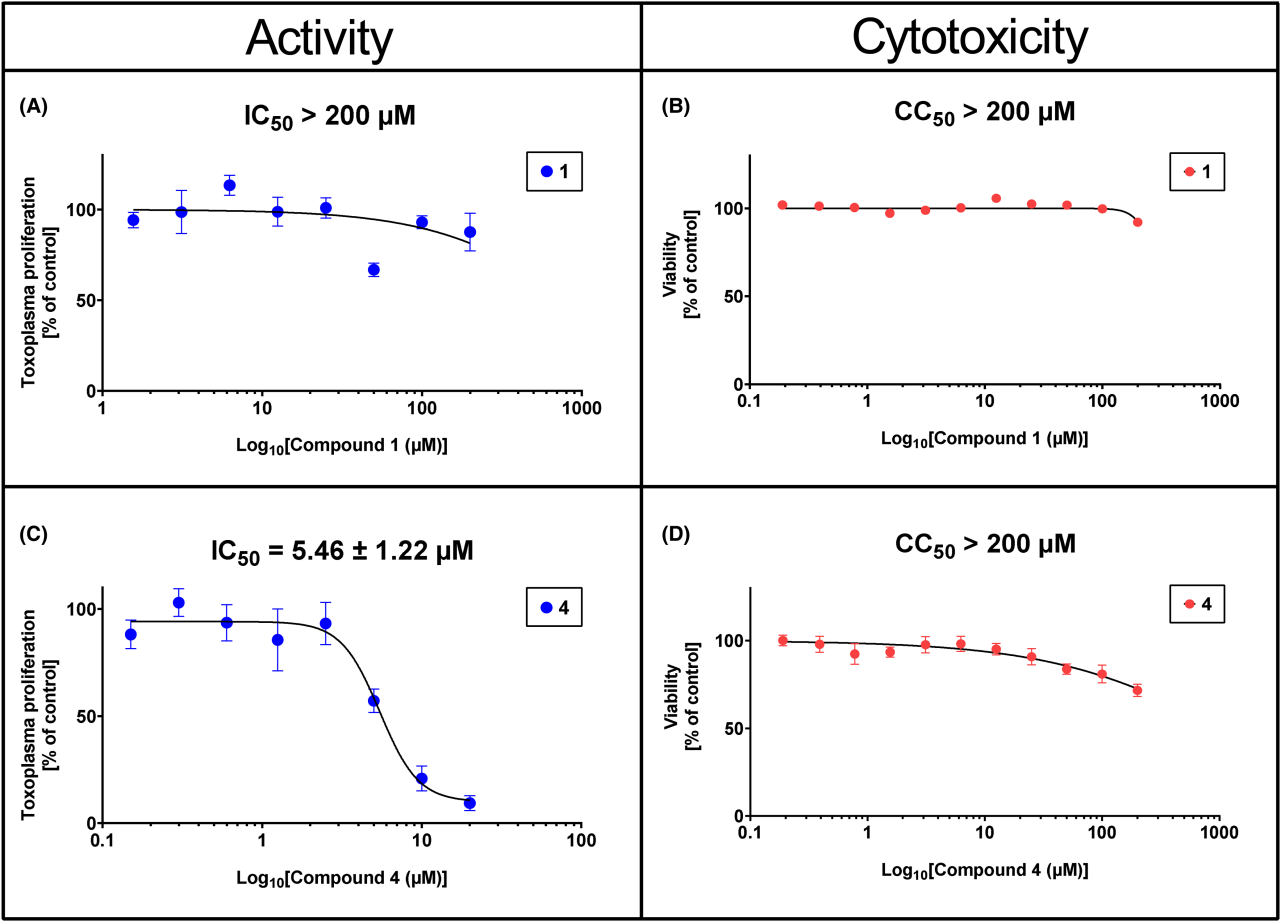
Anti-toxoplasma activity and cytotoxicity on human fibroblasts Hs27 of fosmidomycin and the 3,4-dichlorophenyl-thia analogue. The antiprotozoal activity of 4 (**A**) was determined by the *T. gondii* inhibition assay via the amount of [^3^H]-uracil incorporation into the RNA of the parasite *in vitro*. Cytotoxicity of 4 (**B**) was measured by MTT assays on human fibroblasts Hs27. Data shown are the means of three independent experiments each performed in duplicate (*n* = 6) ± SEM. IC_50_ ± S.D. and CC_50_ values of compound **4** are shown.

To determine if reverse thia and reverse oxa analogues of **1** (Figure 7) could inhibit *T. gondii* proliferation *in vitro*, we conducted an evaluation to assess their anti-parasitic activity and their IC_50_ values against the proliferation of *T. gondii* (type II ME49 strain) with an ^3^[H]-uracil incorporation assay *in vitro*. Interestingly, contrary to **1** that showed no activity accordingly to previous reports [32–37,44]. The *Tg*DXR inhibitors **3**, **4**, **5**, **6**, and **7** demonstrated activity against *T. gondii* growth (Figure 9, Table 2 and Supplementary Figure S6). Moreover, as was first shown in the enzymatic assays, a significant increase in activity for the thia isosters compared with the oxa isoster was observed. Notably, the thia analogue **4** which bears a 3,4-dichlorophenyl moiety in the α-position of the linker demonstrated the most potent inhibitory activity (IC_50_ = 5.46 µM) (Figure 9, Table 2 and Supplementary Figure S6).

**Table 2.** *In vitro* activity of *Tg*DXR inhibitors and pyrimethamine against *T. gondii* ME49 tachyzoites and their cytotoxicity on human fibroblasts Hs27.

Compound	IC_50_ ± S.D. (µM)	CC_50_ (µM)
**1**	>200	>200
**2**	>100	>200
**3**	14.39 ± 1.94	>200
**4**	5.46 ± 1.22	>200
**5**	55.99 ± 6.11	>200
**6**	12.51 ± 5.56	>200
**7**	48.20 ± 8.56	>200
**8**	>100	>200
**9**	>100	>200
**10**	>100	>200
**Pyrimethamine**	0.22 ± 0.05	>100

Values shown in the table represent the means of three independent experiments each done in duplicate (*n* = 6) ± S.D. Pyrimethamine was used as anti-toxoplasma reference compound.

#### Reverse fosmidomycin analogues are not cytotoxic on human host cells

To assess the host cell cytotoxicity of the investigated compounds, MTT assays with Hs27 cells and the *Tg*DXR inhibitors were performed. Similar to **1**, all the reverse thia and oxa analogues did not show any detectable host cytotoxicity at the concentration range of 0.19–200 µM (Figure 9, Table 2 and Supplementary Figure S6). Therefore these *Tg*DXR inhibitors appear to have a good therapeutic index.

#### Molecular docking of compound **4** into *Tg*DXR

**Figure 10.**
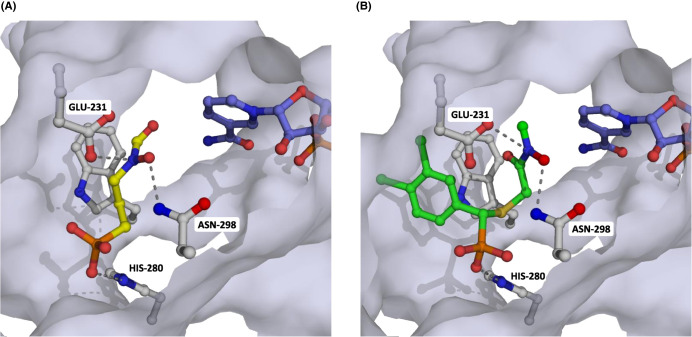
Predicted binding modes of compound 4 in the X-ray crystal structure of *Tg*DXR. (**A**) The fosmidomycin binding site is illustrated, with fosmidomycin represented as yellow sticks, interacting with Glu231, Asn298, and His280. (**B**) Docking results for compound **4** reveal that the 3,4-dichlorophenyl substituent binds in a previously unoccupied pocket.

To elucidate a possible binding mode of compound **4** in *Tg*DXR and to predict which enantiomer of **4** could pose the eutomer, the *S*- and *R*-enantiomer of **4** were docked into the X-ray crystal structure of *Tg*DXR from which fosmidomycin was removed. The docking revealed possible binding modes where the *R*-enantiomer of **4** binds outside the fosmidomycin binding pocket whereas the *S*-enantiomer of **4** binds into the binding pocket (Figure 10). In its predicted binding mode, the *S*-enantiomer of **4** binds similarly to fosmidomycin with the phosphonic acid and the hydroxamic acid groups occupying the same positions. The hydroxamic acid group in **4** however is turned compared with fosmidomycin, which might be a result of the change in direction in which this group is attached to the core of **4**. This, however, does not allow for interactions of the carbonyl group with Asn298 of *Tg*DXR in contrast with fosmidomycin. Yet, **4** places the 3,4-dichlorphenyl substituent in a previously unoccupied pocket, which increases the number of hydrophobic interactions to *Tg*DXR, which are important for increasing the affinity with the enzyme.

## Discussion

Toxoplasmosis is a widespread disease and current treatment regimens are related to adverse effects and additionally, are not able to eradicate the latent phase of the infection. Thus, the identification of novel drug targets is crucial for the development of novel, potent and safer anti-toxoplasma drugs for improving the health of patients at risk of toxoplasmosis [45]. The MEP pathway has been well established as a promising drug target for novel antimicrobial agents, due to its crucial role on the viability of the microorganism and, additionally, its absence in the human host, that allows the development of selective inhibitors [46].

DXR inhibitors are a class of antimicrobial agents studied intensively in *P. falciparum*, *E. coli* and *M. tuberculosis* [34–36]. From previous SAR studies, it has been shown that the retro-inversion of the hydroxamate moiety of **1** and its N-methylation strongly improve the hydrophobic interaction of Trp212 of *Ec*DXR [47] and that the addition of the α-phenyl substitution of the N-methylated reverse fosmidomycin analogues strongly inhibits the growth of the apicomplexan parasite *P. falciparum* [48,49].

DXR inhibitors against *T. gondii* have been already investigated by Cai et al. [37], precisely α-phenyl and α-pyridine substituted fosmidomycin analogues, pyridine containing phosphonate compounds, non-hydroxamate phosphonate inhibitors containing a pyridine and 1-hydroxy-5-phenylpyridin-2-one moiety without a phosphonate group. All the compounds were active against *Tg*DXR, but none of them inhibited the parasite growth, probably due to the permeability barrier posed by parasite plasma membrane(s) of *T. gondii* that is lacking the fosmidomycin transporter GlpT [33].

In this study, we present the first crystal structure of *Tg*DXR in the tertiary complex with NADPH and fosmidomycin. No magnesium ion was observed in this structure despite the fact that it is essential for activity and was included in the crystallization experiments, which also was the case for the *E. coli* DXR protein [23]. The overall structure of *Tg*DXR resembles its counterparts in various species [23,25,40,41]. A search for homologous structures revealed that overall the DXR protein are very similar, with an r.m.s.d. of 1.2–1.8 Å for the overall structure (calculated using the EBI fold server). This shows that the overall fold is conserved. Furthermore, also the amino acids involved in DXP substrate binding and conversion are conserved among the different enzymes. Not surprisingly, also the binding site for fosmidomycin at *Tg*DXR consists of the same amino acids as seen in the structure of the *Ec*DXR, indicating that the domain of *Tg*DXR is similar to the *Ec*DXR domain (Figure 4C). The distance of the hydroxymate region of fosmidomycin to NADPH is 4.4 Å, which is too large for conversion (indicated by the yellow line in Figure 4C). With the substrate DXP, the distance to NADPH is reduced, as an additional bond length is strategically positioned to facilitate the occurrence of the reaction. Since the structure is of medium resolution, we could not identify water molecules with certainty, likely however they play a role in catalysis as shown before [23]. Moreover, the parts of the protein that were not visible in the electron density were determined through SAXS, unveiling their peculiarity. With SAXS however only the position of the C-α atoms can be determined, and atomic detail of the side chains is still lacking which would allow a more detailed description.

**Figure 11.**
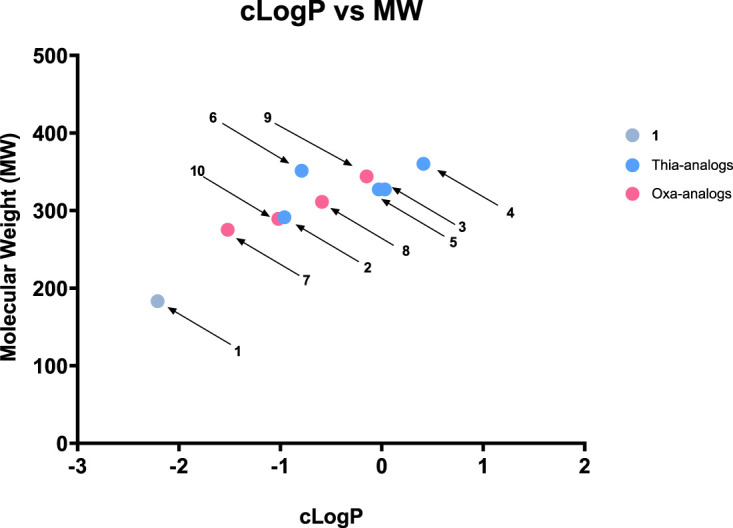
Plot of ClogP vs. MW for fosmidomycin and its thia- and oxa-analogs. Fosmidomycin (**1**) is shown in grey, the thia-analogs in blue, and the oxa-analogs in pink. The investigated fosmidomycin derivatives exhibit increased lipophilicity compared with **1**, with compounds **3** and **4**, the most active compounds, being the most lipophilic. *C*log*P* values have been predicted using the Marvin JS calculator.

In addition, we performed a biological investigation on *Tg*DXR, repurposing already investigated reverse α-phenyl-β-oxa and β-thia-substituted analogues of (**1**) which were previously described as potent inhibitors of *P. falciparum*, *E. coli* and *M. tuberculosis* DXR enzymes [34–36]. We show that all the investigated compounds possess *Tg*DXR inhibitor activity, with the reverse thia analogues presenting a more pronounced activity than the reverse oxa analogues. Moreover, we noticed that inhibitors with a the 3,4-halogen substitution of the phenyl moiety of the reverse thia analogues confers the highest potency against the enzymatic activity of *T*gDXR (Figure 8, Table 1 and Supplementary Figure S5). In the cell-based assays, we demonstrated that the *T*gDXR inhibitors **3**–**7** inhibit *T. gondii* proliferation in contrast with **1** (Figure 9, Table 2 and Supplementary Figure S6). As shown in previous studies, *T. gondii* is unaffected by the activity of compound (**1**) probably due to its lack of the GlpT transporter, the glycerol-3-phosphate (G3P) transporter responsible for the drug uptake of (**1**) in *E. coli* [31,33]. *T. gondii* does not possess a protein with significant sequence similarity to *E. coli* GlpT. This suggests that the activity of the derivatives of (**1**) on *T. gondii* may be due to the use of a different transporter protein or their increased lipophilicity, as indicated by their higher ClogP values compared with (**1**) in Figure 11, that could facilitate passive transport through the membranes [50].

*Tg*PiT and *Tg*PT2 are plasma membrane transporters found in *T. gondii*. Notably, *Tg*PT2, which is only present in coccidian parasites, is essential for parasite growth. These proteins, possessing phosphate transport activities [51], could be potential candidates for transporting reverse fosmidomycin derivatives.

Additionally, we observed once again a trend within the thia analogues, showing higher potency compared with the cognate oxa analogues. Previous studies showed that the increased activity of the α-phenyl-β-thia-substituted analogues against *Ec*DXR and *Mt*DXR enzymes is attributed to the interaction of the sulfur atom with the conserved Met298 within the flexible loop of the enzymes [34,48,50]. Furthermore, in this context, the 3,4-dichlorophenyl substituted compound **4** emerged as the most potent one in inhibiting *T. gondii* proliferation, with an IC_50_ of 5.46 µM, presumably due the improved lipophilicity provided by the 3,4-dichlorophenyl substitution (Figure 11) that enhances cellular uptake [52].

## Conclusions

In summary, in the present study we report the first X-ray structure of *Tg*DXR co-crystallized with fosmidomycin and the cofactor NADPH and its biochemical characterization, as well as the identification of reverse α-phenyl-β-thia- and β-oxa-isosters of fosmidomycin as novel anti-*T. gondii* agents with in part potent *Tg*DXR inhibitory activity*.* The ternary co-crystal structure of *Tg*DXR and **1** will serve as starting points for the design and development of improved *Tg*DXR inhibitors. Further efforts are needed for the development of enhanced derivatives and to explore the efficacy *in vivo*.

## Material and methods

### Biochemical characterization

#### Sequence alignment

The amino acid sequence of the investigated protein *Tg*DXR (NCBI Reference Sequence: XP_018635719.1) was compared with a multiple sequence alignment using Clustal Omega sequence analysis tool (https://www.ebi.ac.uk/Tools/msa/clustalo/) [53] with its default settings with DXR enzymes from other species that have been extensively studied: *P. falciparum* DXR (*Pf*DXR, NCBI Reference Sequence: AAD03739.1), *M. tuberculosis* DXR (*Mt*DXR, NCBI Reference Sequence: OHO19719.1) and *E. coli* DXR (*Ec*DXR, NCBI Reference Sequence: WP_302347400.1) and visualized and the percentage of identity analysed with Jalview software version 2.11.2.7 [54] (Supplementary Figure S1).

#### *Tg*DXR

The gene of 1-Deoxy-d-xylulose 5-phosphate reductoisomerase of *T. gondii* (*Tg*DXR) ME49 strain was identified using *ToxoDB database (*http://ToxoDB.org). The gene of *Tg*DXR (NCBI: NC_031478.1), optimized for expression in *E. coli*, was obtained from GenScript (Piscataway, Township, NJ, U.S.A.) as a synthetic gene subcloned into the pET-16b plasmid, using NdeI/BamHI restriction sites. The resulting expressed protein carries an N-terminal decahistidine tag (His_10_). However, the purification of the recombinant His_10_-tagged *Tg*DXR proved to be challenging. To overcome these issues and to generate a soluble recombinant protein, another expression plasmid was designed. We deleted 181 amino acids (2–182) of the *Tg*DXR sequence representing the bipartite apicoplast targeting peptide [37] in order to generate a soluble truncated His_10_-tagged *Tg*DXR containing the sequence of the catalytic domain (Supplementary Figure S1).

#### Cloning of truncated His_10_-*Tg*DXR and construction of the expression plasmid

The generation of the truncated His_10_-*Tg*DXR commenced with the deletion of 181 amino acids via Site-Directed-Mutagenesis method [55]. For this purpose, the coding sequence of the truncated *Tg*DXR was amplified from the pET-16b plasmid construct with a polymerase chain reaction (PCR) (Phusion® High-Fidelity PCR Kit, New England BioLabs, Frankfurt am Main, Germany, #E0553S) using the primers *Tg*DXR-del181AA_For and *Tg*DXR-del181AA_REV (Supplementary Table S1). Then, the coding sequence was purified through an 1% agarose gel purification [56] and ligated with a KLD- Enzyme Mix (KLD Enzyme Mix, New England BioLabs, Frankfurt am Main, Germany, #M0554S). Subsequently the plasmids were transformed into an *E. coli* DH5α (DH5α Competent Cells, Thermo Fisher Scientific, Waltham, MA, U.S.A., #EC0112), which then were streaked onto Luria-Bertani (LB) agar-plates containing ampicillin (100 µg/ml) (Ampicillin, Thermo Fisher Chemicals, Thermo Fisher Scientific, Waltham, MA, U.S.A., #J60977.14). To confirm the successful cloning, positive clones were identified through PCR. Additionally, DNA sequencing was performed using T7 forward and reverse primers for further verifications. The *Tg*DXR expression plasmid was extracted with a plasmid miniprep kit (Monarch® Plasmid Miniprep Kit, New England BioLabs, Frankfurt am Main, Germany, #T1010L).

#### Expression of truncated His_10_-*Tg*DXR

The expression plasmid *Tg*DXR was transformed into the chemically competent *E. coli* BL21 (DE3) cells (BL21 (DE3) Singles™ Competent Cells — Novagen, Merck KGaA, Darmstadt, Germany, #70235). Then *E. coli* transformants were streaked onto LB agar-plates containing ampicillin (100 µg/ml) (Ampicillin, Thermo Fisher Chemicals, Thermo Fisher Scientific, Waltham, MA, U.S.A., #J60977.14) and incubated overnight at 37°C.

To express the His-tagged protein, 100 ml overnight pre-cultures were prepared using fresh colonies from LB agar plates or 50% glycerol cryo stock stored at −80°C. The day after, 1 l of freshly prepared and autoclaved LB media (10 g Triptone, 5 g yeast extract and 5 NaCl) was supplemented with 100 μg/ml of ampicillin and inoculated with the pre-cultures to an optical density at 600 nm (OD_600_) of 0.1. The main culture was then incubated with shaking at 37°C and 180 rpm. At an OD_600_ of 0.6, protein expression was induced with the supplementation in the culture of isopropyl-β-d-thiogalactopyranosid (IPTG, Merck KGaA, Darmstadt, Germany, #I6758) to a final concentration of 1 mM. The main culture was further incubated for additional 2 h with shaking at 37°C and 180 rpm. Afterwards, cells were harvested via centrifugation at 5000*g*, for 15 min at 4°C (rotor SLC-6000, Sorvall, Thermo Fischer, Waltham, MA, U.S.A.), the supernatant discarded. The cell pellet was used subsequently or snap frozen in liquid nitrogen and short-term stored at −20°C.

#### Purification of truncated His_10_-*Tg*DXR

Truncated His_10_-*Tg*DXR purification involved two main steps: immobilized metal ion affinity chromatography (IMAC) and SEC.

Cells were thawed at 4°C and suspended with ice-cold lysis buffer A (50 mM NaH_2_PO_4_, 300 mM NaCl, 20 mM imidazole, pH 8) supplemented with protease inhibitor cocktail (cOmplete Protease Inhibitor Cocktail, Roche, Basel, Switzerland). Subsequently, cell lysis was performed through the application of shear force using a cell disruptor/homogenizer (M-110P Microfluidizer, Microfluidics Inc., Westwood, MA, U.S.A.). This method was employed to effectively break down the cells and release their content. To eliminate cell membranes and other insoluble detritus, ultracentrifugation of the lysate was performed at 100,000*g*, for 45 min at 4°C (Beckman Optima XE, Beckman Coulter Inc., Brea, CA, U.S.A.). Then, the collected supernatant was purified via Ni-IMAC at 4°C on a Ni^2+^ pre-treated HiTrap IMAC FF 5 ml column (Cytiva Life Science, Marlborough, MA, U.S.A., #17092104) using a protein purification system (Äkta Purifier 10, GE Healthcare, Chicago, IL, U.S.A.). The protein was loaded on the column with a flow rate of 1 ml/min. After binding, the resin was washed with buffer B (50 mM NaH_2_PO_4_, 150 mM NaCl, 20 mM imidazole, pH 8) to remove the non-specifically bound proteins. Afterwards, the protein was eluted with a gradually increasing concentration of imidazole with buffer C (50 mM NaH_2_PO_4_, 150 mM NaCl, 300 mM imidazole, pH 8). As determined by absorption at *λ* 280 nm, the fractions containing proteins were then pooled, concentrated, and subjected to a SEC to further improve its purity. SEC was performed on an Äkta Purifier 10 equipped with a pre-equilibrated Superdex 200 Increase 10/300 GL column (Cytiva Life Science, Marlborough, MA, U.S.A., #28990944) at a flow rate of 0.5 ml/min with buffer D (20 mM Tris–HCl, 150 mM NaCl, 2% Glycerol, pH 7.5). Dimeric protein was collected, concentrated, aliquoted, and snap-frozen for storage at −80°C.

#### Determination of protein concentration

The concentration of the protein was assessed by measuring its absorbance with a microvolume UV-Vis spectrophotometer (NanoDrop™ One, Thermo Fisher Scientific, Waltham, MA, U.S.A.) using protein-specific parameters, including the molecular mass (51,808 Da) and the extinction coefficient of the protein (34,420 M^−1^ cm^−1^) obtained with the web-software tool Expasy ProtParam [57].

#### *T. gondii* DXR enzyme kinetic characterization and optimization

To biochemically characterize the catalytic ability of the truncated *Tg*DXR for the conversion of DXP into MEP in the presence of the cofactors Mg^2+^ and NADPH, and to determine the optimum conditions for maximum enzyme activity, the enzymatic assays were monitored at 340 nm (maximal absorbance of NADPH) in different conditions.

The enzymatic activity was evaluated in fixed conditions, using 50 mM HEPES buffer containing 50 µg/ml of bovine serum albumin (BSA) containing 100 nM of purified *Tg*DXR protein in dimeric state. In each experimental evaluation, only one parameter was varied: either the substrate (DXP) concentration (ranging from 7.8 to 500 µM), or the cofactor NADPH (ranging from 7.8 to 500 µM), or Mg^2+^ concentration (ranging from 1 to 6 mM) or the pH (ranging from 5.5 to 9) (Figure 3).

For the determination of the kinetic parameters, various concentration of the substrate DXP or NADPH were employed for the determination of the apparent *K_m_* (substrate concentration that yield a half-maximal velocity) and *V*_max_ (maximum velocity) for the enzyme. These parameters were calculated by a non-linear regression with the software GraphPad PRISM™ (Version 9.5.1; San Diego, CA, U.S.A.) plotting the initial velocity of the reactions and using the Michaelis–Menten model (Figure 3).

#### Site-directed mutagenesis and expression of *Tg*DXR mutants

Site-directed mutagenesis of the truncated His_10_-*Tg*DXR was performed on the pET16-b plasmid (see above) using the Site-Directed-Mutagenesis method in order to generate Glu231Ala, His280Ala, and Asn298Ala mutants. Briefly, the coding sequence of truncated *Tg*DXR was amplified from the pET-16b plasmid using PCR with primers reported in Supplementary Table S2. The amplified sequence was purified via 1% agarose gel and ligated using KLD Enzyme Mix. The plasmids were then transformed into *E. coli* DH5α cells and streaked onto LB agar plates with ampicillin (100 µg/ml). Positive clones were confirmed by PCR and further verified by DNA sequencing using T7 primers. Finally, the *Tg*DXR expression plasmid was extracted using the Monarch^®^ Plasmid Miniprep Kit. The mutant *Tg*DXR plasmids were used to transform *E. coli* BL21 (DE3) competent cells for expression under conditions identical to those used for wild-type expression. Cells were cultured at 37°C in LB medium with shaking until reaching an optical density at 600 nm of 0.6. Protein expression was induced with 1 mM IPTG, followed by an additional 3-h incubation at 37°C. Cells were harvested by centrifugation (5000*g* for 15 min at 4°C) and stored at −80°C until needed. The histidine-tagged proteins were subsequently purified as described for the wild-type protein (see section ‘Purification of truncated His_10_-*Tg*DXR’). Structural characterization of the *Tg*DXR catalytic domain.

#### Crystallization and structure determination of *Tg*DXR

*Tg*DXR was crystallized by sitting-drop vapor-diffusion at 12°C at a concentration of 7–10 mg/ml with 20 mM TRIS pH 7.5, 150 mM NaCl, 2% glycerol containing 1 mM fosmidomycin, 4 mM MgCl_2_ and 4 mM NADPH. Crystals formed after a couple of days, were cryo-protected with 20% ethylene glycol, washed in mineral oil and flash frozen in liquid nitrogen. Diffraction data were collected at 100 K at beamline ID30-B (ESRF, Grenoble, France) using a 0.9793 Å wavelength. Data reduction was performed using XDS [58] and aimless [59] from the CCP4 suite [60]. The structure was solved via molecular replacement with Phaser [61]. The initial model was refined alternating cycles of manual model building in COOT [62,63] and automatic refinement using Phenix [64] version 1.19.2_4158. Data collection and refinement statistics are reported in Supplementary Table S2.

#### SAXS from *Tg*DXR

We collected the SEC-SAXS data on beamline BM29 at the ESRF Grenoble [65,66]. The BM29 beamline was equipped with a PILATUS 2M detector (Dectris) at a fixed distance of 2.827 m.

The SEC-SAXS measurement was performed at 10°C with a *Tg*DXR protein concentration of 8.00 mg/ml. The SEC-SAXS run was performed on a Superdex 200 increase 10/300 column (300 µl inject, buffer: 20 mM Tris–HCl, 150 mM NaCl, 40 mM MgCl_2_, 2% glycerol, pH 7.5) with a flowrate of 0.5 ml/min. We collected 1500 frames with an exposer time of 2 s/frame and scaled the data to absolute intensity against water.

All used programs for data processing were part of the ATSAS Software package (Version 3.0.5) [67]. Primary data reduction was performed with the programs CHROMIXS [68] and PRIMUS [69]. With the Guinier approximation [70], we determined the forward scattering *I*(0) and the radius of gyration (*R_g_*). The program GNOM [71] was used to estimate the maximum particle dimension (*D*_max_) with the pair-distribution function *p*(*r*). We used the partially solved crystal structure as template in an EOM [72,73] and added the missing amino acids from the loop region and the N-terminus to each protomer.

#### Molecular docking of compound **4** into *Tg*DXR

For the molecular docking the two enantiomers of compound **4** were drawn and converted into 3D using the ChemDraw19 suite. The enantiomers of **4** were subsequently docked into the X-ray crystal structure of DXR from which **1** was removed utilizing a combination of AutoDock as a docking engine and the DrugScore2018 distance-dependent pair-potentials as an objective scoring function [74,75]. During docking, default parameters were used, except for the clustering RMSD cut-off, which was set to 2.0 Å [74–76]. The docking grids were centred on the **1** binding site. Binding modes were considered valid if they were contained in the largest cluster with the most favourable docking energies, which comprised at least 20% of all docking poses.

#### Visualization and analysis of molecular structures

For figure preparation of the crystal structures of *Tg*DXR enzyme we utilized PyMOL software suite (www.pymol.org) [77].

### *In vitro* biological evaluation

#### Compounds

Fosmidomycin (**1**) (Fosmidomycin sodium salt, Invitrogen, Thermos Fisher Scientific, Waltham, MA, U.S.A., #FR-31564) was dissolved in Dulbeccós Phosphate Buffered Saline ((1x), Gibco-Thermo Fisher Scientific, Waltham, MA, U.S.A., #14190144). DXR inhibitors (((2-(hydroxy(methyl)amino)-2-oxoethyl)thio)(phenyl)methyl)phosphonic acid (**2**); ((3,4-difluorophenyl)((2-(hydroxy(methyl)amino)-2-oxoethyl)thio)methyl)phosphonic acid (**3**), ((3,4-dichlorophenyl)((2-(hydroxy(methyl)amino)-2-oxoethyl)thio)methyl)phosphonic acid (**4**), ((3,5-difluorophenyl)((2-(hydroxy(methyl)amino)-2-oxoethyl)thio)methyl)phosphonic acid (**5**), ((3,5-dimethoxyphenyl)((2-(hydroxy(methyl)amino)-2-oxoethyl)thio)methyl)phosphonic acid (**6**), ((2-(hydroxy(methyl)amino)-2-oxoethoxy)(phenyl)methyl)phosphonic acid (**7**), ((3,4-difluorophenyl)(2-(hydroxy(methyl)amino)-2-oxoethoxy)methyl)phosphonic acid (**8**), ((3,4-dichlorophenyl)(2-(hydroxy(methyl)amino)-2-oxoethoxy)methyl)phosphonic acid (**9**), ((2-(hydroxy(methyl)amino)-2-oxoethoxy)(p-tolyl)methyl)phosphonic acid (**10**) employed in this study (Figure 7) were prepared using procedures from previously published methods [34–36] and dissolved in DMSO (dimethyl sulfoxide, ≥99%, Thermo Scientific Chemicals, Waltham, MA, U.S.A., #A12380.36). Staurosporine (Merck, Darmstadt, Germany, #S4400) and pyrimethamine (Merck, Darmstadt, Germany, #219864) were dissolved in DMSO. All the compounds were prepared as 10 mM stock solutions and stored at −20°C. Before use, these solutions were thawed and diluted in culture medium to produce the appropriate concentrations (ranging from 0.0003 to 200 µM).

#### *T. gondii* DXR enzyme inhibition assays

The enzymatic assays were conducted at 30°C in 96 well plates using a total reaction volume of 150 µl containing 100 nM of purified *Tg*DXR protein in dimeric state, 100 µM of NADPH and 4 mM of MgCl_2_ as cofactors, 100 µM of DXP as substrate in 50 mM HEPES buffer (pH 7.5) containing 50 µg/ml of BSA. For the screening, DXR inhibitors were tested for their inhibitory activity and their IC_50_ measurements at concentrations ranging from 100 µM to 3.05 nM, in dilution steps 1:2.

To optimize and to ensure the interaction of NADPH and its enzyme binding pocket, the assay solution was incubated for 10 min at 37°C. Then, the reaction was commenced with the addition of 100 µM of DXP to the complete assay mixture. The reaction was monitored by measuring the absorption at 340 nm every minute for 1 h using a microplate reader (Tecan® 200 Pro, Tecan Group, Männedorf, Switzerland). The initial velocity of the reactions were calculated, the values were then used in the software GraphPad PRISM™ (Version 9.5.1; San Diego, CA, U.S.A.) for determinations of the IC_50_ values. The inhibitor constant values (*K_i_*) of all inhibitors were determined using the Michaelis–Menten formula *K_i_* = IC_50_/(1 + [*S*]/*K_m_*), [*S*] is the concentration of DXP (100 μM) and *K_m_* was calculated as 30.58 μM.

#### Enzymatic assays of *Tg*DXR mutants Glu321Ala, His280Ala and Asn298Ala

After purifying the *Tg*DXR mutants Glu321Ala, His280Ala and Asn298Ala, their activities were tested in comparison with the *Tg*DXR wildtype. Enzymatic assays were conducted under the same conditions described in section ‘*T. gondii* DXR enzyme inhibition assays’.

#### *T. gondii* and host cells *in vitro* culture

*T. gondii* tachyzoites of the ME49 strain (ATCC/LGC Standards GmbH, Wesel, Germany, #50611), were cultured and maintained by repeat passage in monolayers of human foreskin fibroblasts Hs27 (ATCC/LGC Standards GmbH, Wesel, Germany, #CRL-1634) as host cells. Cultures were grown in Iscove's modified Dulbecco's medium (IMDM; Gibco-Thermo Fisher Scientific, Waltham, MA, U.S.A., #12440053) supplemented with 10% heat-inactivated fetal bovine serum (FBS Standard; South America origin, FBS, 2 µm sterile filtered, PAN-Biotech, Aidenbach, Germany, #P30-3306) and 50 mM 2-mercaptoethanol (Gibco-Thermo Fisher Scientific, Waltham, MA, U.S.A., #21985023) at 37°C and 5% CO_2_ as previously described [78–80].

#### *T. gondii in vitro* growth assay

With the aim to assess the inhibitory *in vitro* effect on parasite growth, *T. gondii* growth assays were performed as described previously [78–80]. Compounds, previously diluted in culture media in appropriate stock solutions, were added to confluent monolayers of Hs27 cells in 96-flat well plates at various concentrations. Then, freshly harvested tachyzoites were added to the cultures at a multiplicity of infection of 1:1 (parasite/host cell ratio). As controls, untreated and uninfected Hs27 cells, 24 h pre-stimulated and *T. gondii* infected cells with human interferon γ (IFN γ) (300 U/ml) (Merck, Darmstadt, Germany, #I17001) and *T. gondii* infected cells only were employed. After 48 h incubation, proliferating toxoplasmas were labelled with 0.3 μCi/well of tritiated uracil (^3^H-U; 5 mCi, Hartmann Analytic, Braunschweig, Germany, #ART1782) [81]. After 28–30 h of incubation, plates were frozen at −20°C overnight. Cells were then thawed and harvested (Basic96 Harvester; Zinsser Analytic, Skatron Instruments, Northridge, CA, U.S.A.), purified with glass fibre filters (Printed Filtermat A 102 mm × 258 mm; PerkinElmer, Waltham, MA, U.S.A.) and dried at 130°C for 15–20 min. Dried filters were then wrapped into transparent plastic covers, dunked with 10 ml scintillation cocktail (Betaplate Scint; PerkinElmer, Waltham, MA, U.S.A., #1205-440), sealed and then loaded into metal cassettes and the incorporation of ^3^H-uracil into the RNA of proliferating toxoplasmas was measured using a β-counter, a liquid scintillation counter that measure the Cherenkov radiation (Betaplate Liquid Scintillation Counter 1205; LKB-WALLAK, Melbourne, Australia). All data sets were normalized to 100% of the positive control. The dose-response curves of the compounds were fitted by means of the statistics software GraphPad PRISM™ (Version 9.5.1; San Diego, CA, U.S.A.). The minimal concentrations of compounds required for 50% inhibition of the parasite (IC_50_ values) were determined by non-linear regression analysis. Pyrimethamine [13,82] was used as reference anti-toxoplasma compound.

#### Cytotoxicity assays

The method based on the reduction of the tetrazolium dye MTT [3-(4,5-dimethylthiazole-2-yl)-2,5-diphenyltetrazolium bromide] to its purple insoluble formazan by mitochondrial NAD(P)H-dependent cellular oxidoreductase enzymes in living cells, previously described by Mosmann [83] was employed to assess the cytotoxic effects of the examined DXR inhibitors on the host cells, as previously described [78–80]. Briefly, Hs27 cells were cultured in 96-well plates at a concentration of 5 × 10^4^/well in (IMDM, Gibco–Thermo Fisher Scientific, Waltham, MA, U.S.A., #12440053) with a volume of 100 µl per well and incubated at 37°C overnight. Then, eleven two-fold serially diluted concentrations of the investigated compounds ranging from 200 to 0.09 µM were prepared and added to each well. Hs27 cells were then incubated for 24 h in humidified atmosphere of 5% CO_2_ in air. Controls included wells containing medium alone without cells (blank), untreated Hs27 cells, DMSO (negative control) and staurosporine (0.031, 0.062, 0.125, 0.25, 0.5, 1 µM) (Merck, Darmstadt, Germany, #S4400) as apoptosis inducer [84]. After incubation, the culture media was replaced with 100 µl of DMEM medium without phenol (Gibco-Thermo Fisher Scientific, Waltham, MA, U.S.A., #21041025) plus 10% heat-inactivated (FBS Standard, South America origin, FBS, 2 µm sterile filtered, PAN-Biotech, Aidenbach, Germany, #P30-3306), and 50 mM 2-mercaptoethanol (Gibco-Thermo Fisher Scientific, Waltham, MA, U.S.A., #21985023). Thereafter, the MTT reagent was added to each well and analysis conducted following the manufacture instructions (CyQuant MTT Cell Viability Assay Kit, Thermo Fisher Scientific, Waltham, MA, U.S.A., #V-13154). The absorbance was measured at 570 nm on an ELISA microplate reer (TECAN Sunrise, Männedorf, Switzerland) and the percentage of viability was calculated when compared with the untreated control. The data set was subsequently adjusted by applying blank correction. Cell viability values expressed as percentage of the negative control value were calculated as follows:

$$\text{\%}\;\;\;\mathrm{Cell}\;\;\mathrm{viability}=\frac{\mathrm{mean}\;\;\mathrm{absorbance}\;\mathrm{of}\;\;\mathrm{treated}\;\mathrm{wells}-\mathrm{blank}}{\mathrm{mean}\;\;\mathrm{absorbance}\;\;\mathrm{of}\;\mathrm{negative}\;\mathrm{control}-\mathrm{blank}\;}\times 100.$$

The cytotoxicity of each compound was expressed as half maximal cytotoxic concentration (CC_50_ values) against Hs27 cells. These values were calculated by a non-linear regression using GraphPad PRISM™ statistics software package (Version 9.5.1; San Diego, CA, U.S.A.) plotting the percentage viability against the log of compound concentrations.

## Data Availability

All supporting data are included within the main article and its supplementary files. We uploaded the SAXS data to the Small Angle Scattering Biological Data Bank (SASBDB) [85], with the accession codes SASDS47. The structure of *Tg*DXR has been deposited in the Protein Databank (PDB) under accession code: 8S65.

## Abbreviations

BSA: bovine serum albumin
DMSO: dimethyl sulfoxide
DXP: 1-Deoxy-d-xylulose 5-phosphate
EOM: Ensemble Optimisation Method
FBS: fetal bovine serum
IMDM: Iscove's Modified Dulbecco's medium
IPTG: *i*sopropyl-β-d-thiogalactopyranosid
LB: Luria-Bertani
MEP: 2-*C*-methyl-d-erythritol 4-phosphate
PCR: polymerase chain reaction
SAXS: small-angle X-ray scattering
SEC: size exclusion chromatography
