# Supplementary material for: 1-Deoxy-d-xylulose 5-phosphate reductoisomerase as target for anti Toxoplasma gondii agents: crystal structure, biochemical characterization and biological evaluation of inhibitors
Source: Biochem J. 2024 Aug 21;481(16):1075–96. doi: 10.1042/BCJ20240110 (PMC11346426; doi:10.1042/BCJ20240110)
Supplement: Supplementary Material [file BCJ-481-1075-s1.pdf]

Supplementary Information

1-deoxy-D-xylulose 5-phosphate reductoisomerase (DXR) as target for anti *Toxoplasma gondii* agents: crystal structure, biochemical characterisation and biological evaluation of inhibitors

Flaminia Mazzone[1]*, Astrid Hoeppner[2], Jens Reiners[2], Christoph G.W. Gertzen[2,3] Violetta Applegate[2], Mona A. Abdullaziz[3,4], Julia Gottstein[5], Daniel Degrandi[1], Martina Wesemann[5], Thomas Kurz[3]†, Sander H. J. Smits[2,5]†* and Klaus Pfeffer[1]†*

[1]Institute of Medical Microbiology and Hospital Hygiene, Heinrich Heine University, Düsseldorf, University Hospital Düsseldorf, Germany

[2]Center for Structural Studies, Heinrich Heine University, Düsseldorf, Germany

[3]Institute of Pharmaceutical and Medicinal Chemistry, Heinrich Heine University, Düsseldorf, Germany

[4]National Research Centre (NRC), Dokki, Cairo, Egypt

[5]Institute of Biochemistry, Heinrich Heine University, Düsseldorf, Germany

†These authors share last authorship

*Authors for correspondence:

Klaus Pfeffer
klaus.pfeffer@hhu.de,

Sander H.J. Smits
sander.smits@hhu.de,

Flaminia Mazzone
flaminia.mazzone@hhu.de

1 Multiple sequence alignment

Previous studies on *P. falciparum, E. coli*, and *M. tuberculosis* showed that the DXR enzyme is the biological target of the reverse thia and oxa analogues employed in this study (1-3). The DXR enzymes of these species have been extensively studied (1, 4-6), but very less is known for *T. gondii* DXR (7). *Tg*DXR shares a high degree of sequence similarity with DXRs from other species (7), resulting in an highly conserved catalytic domain among all species in comparison (**Figure S1**). The *Tg*DXR sequence is composed of 632 residues. The initial 186 amino acids from the N-terminal region (1 – 186) represent the bipartite apicoplast targeting peptide, since this extension in only present in the apicomplexan parasites *T. gondii* and *P. falciparum* (7, 8). The NADPH binding domain including amino acids 187 – 342 and the metal/substrate binding domain (405 – 632) proved to be highly conserved. Special feature of the *Tg*DXR is the linker region ranging from amino acids (343 – 404). Apart of this region, the amino acids involved in direct contact with the NADPH ligand and substrate are strictly conserved (**Figure S1**).

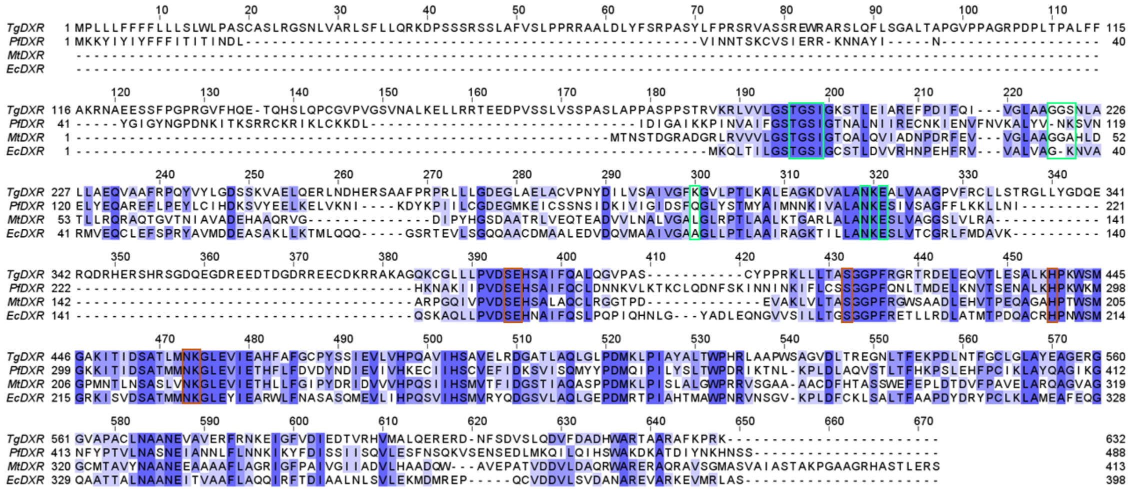

Supplementary Figure S1. Multiple sequence alignment of the amino acids sequence of the putative *T. gondii* DXR

*Tg*DXR, *T. gondii* (NCBI Reference Sequence: XP_018635719.1); *Pf*DXR, *P. falciparum* (NCBI Reference Sequence: AAD03739.1); *Mt*DXR, *M. tuberculosis* (NCBI Reference Sequence: OHO19719.1) and *Ec*DXR, *E. coli* (NCBI Reference Sequence: WP_302347400.1). Identical amino acids are shaded in dark blue, similar amino acids in lighter shades. *Tg*DXR

residues are highlighted according to their function: residues interacting with the NADPH cofactor are highlighted in green, those binding the inhibitor **1** are highlighted in orange. Alignment coloured using Jalview 2.11.2.7.

2 Enzyme production

Supplementary Table S1. List of the primers used in this work and their parameters

Primer name	Sequence	CG %	Tm
TgDXR-del181AA_For	TCCACGCGTGTGAAGAGACTTGTGG	56	75.1
TgDXR-del181AA_Rev	CATATGACGACCTTCGATATGGCCGCTG	53.5	76.6
T7 promoter primer (5')	TAATACGACTCACTATAGGG	40	53.2
T7 terminator primer (3')	GCTAGTTATTGCTCAGCGG	47	54.5

Supplementary Table S2. List of the primers used for the production of the E231A, H280A and N298A mutants of His_{10} *Tg*DXR and their parameters

Primer name	Sequence	CG %	Tm
E231A_for	TGTCGACTCCGCGCACTCGGCAA	65.2	75.1
E231A_rev	GGAAGAAGGAGGCCGCATTTCTGCC	60	72.4
H280A_for	TGCTCTCAAAGCGCCCAAGTGGAGC	60	73.9
H280A_rev	CTTTCAAGAGTTACTTGCTCCAGTTCGTCTCGC	48.4	72.3
N298A_for	AAGTCATTGAAGCTCACTTCGCCTTCGGG	51.7	72.9
N298A_rev	CCAGGCCCTTCGCCATCAACGTC	65.2	72.5

3 Crystal structure parameters and refinement

Supplementary Table S3. Data collection and refinement statistics.

	***Tg*DXR**
**Wavelength**	0.91677
**Resolution range**	54.97 - 2.56 (2.651 - 2.56)
**Space group**	P 65
**Unit cell**	159.524 159.524 75.873 90 90 120
**Total reflections**	67947 (6688)
**Unique reflections**	35325 (3503)
**Multiplicity**	1.9 (1.9)
**Completeness (%)**	99.04 (99.54)
**Mean I/sigma(I)**	11.53 (1.17)
**Wilson B-factor**	65.83
**R-merge**	0.03397 (0.4448)
**R-meas**	0.04804 (0.629)
**R-pim**	0.03397 (0.4448)
**CC1/2**	0.999 (0.76)
**CC***	1 (0.929)
**Reflections used in refinement**	35299 (3500)
**Reflections used for R-free**	1999 (197)
**R-work**	0.1941 (0.3214)
**R-free**	0.2260 (0.3795)
**CC(work)**	0.966 (0.837)
**CC(free)**	0.960 (0.804)
**Number of non-hydrogen atoms**	6416
**macromolecules**	6283
**ligands**	124
**solvent**	9

**Protein residues**	821
**RMS(bonds)**	0.022
**RMS(angles)**	2.03
**Ramachandran favored (%)**	96.43
**Ramachandran allowed (%)**	2.95
**Ramachandran outliers (%)**	0.62
**Rotamer outliers (%)**	0.31
**Clashscore**	18.58
**Average B-factor**	81.21
**macromolecules**	81.05
**ligands**	90.29
**solvent**	66.19

Statistics for the highest-resolution shell are shown in parentheses.

4 *Tg*DXR SEC-SAXS data

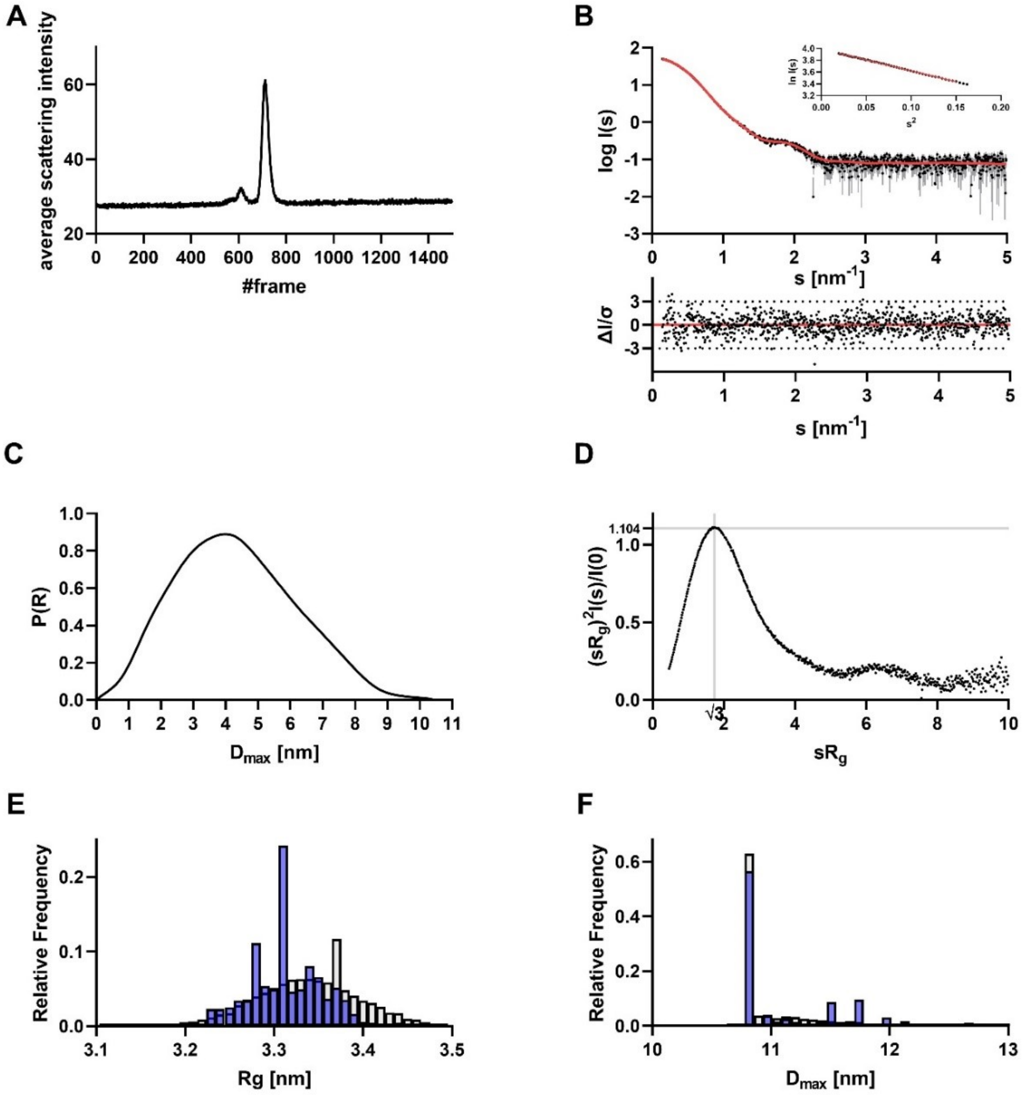

Supplementary Figure S2. Small-angle X-ray scattering data from *Tg*DXR apo.

A: CHROMIXS SEC SAXS elution profile. Each frame corresponds to 2 sec exposer time. **B**: Scattering data of *Tg*DXR. Experimental data are shown in black dots, with grey error bars. The EOM ensemble model fit is shown as red line and below is the residual plot of the data. The Guinier plot of *Tg*DXR is added in the right corner. **C**: *p(r)* function of *Tg*DXR apo offers a D_{max} values of 10.44 nm. **D**: Dimensionless Kratky plot of *Tg*DXR apo showed a compact practical. **E & F**: R_g and D_{max} distribution of *Tg*DXR apo. Ensemble pool is shown in grey, selected EOM models are shown in blue.

4.1 EOM: Ensemble Optimisation Method

Protein sequence (monomer) used for EOM.

Black parts are solved in the crystal and were extracted and used as rigid body. Missing amino acids are shown in green. These were added and orientated from EOM until the models describes the scattering data.

MGHHHHHHHHHHSSGHIEGRHMSTRVKRLVVLGSTGSIGKSTLEIARE-
FPDIFQIVGLAAGGSNLALLAQVAAFRPQYVYLGDSSKVAELQERLNDHERSAAFPRP
RLLLGDEGLAELACVPNYDILVSAIVGFKGVLPTLKALEAGKDVA-
LANKEALVAAGPVFR-
CLLSTRGLLYGDQERQDRHERSHRSGDQEGDREEDTDGDRREECDKRRAKAGQKCG
LLLPVDSEHSAIFQALQGVPASCYPPRKLLLTASGG-
PFRGRTRDELEQVTLESALKHPKWS-
MGAKITIDSATLMNKGLEVIEAHFAFGCPYSSIEVLVHPQAVIHSAVELRDGATLAQL
GLPDMKLPIAYALTWPHRLAAPWSAGVDLTREGNLTFEK-
PDLNTFGCLGLAYEAGERGGVAPACLNAANEVAVERFRNKEIGFVDIEDTVRHVMA
LQERERDNFSDVSLQDVFDADHWARTAARAFKPR

Supplementary Table S4. Overall SAXS Data

**Data collection parameters**	
SAXS Device	BM29, ESRF Grenoble (9, 10)
Detector	PILATUS 2 M
Detector distance (m)	2.827
Beam size	200 µm x 200 µm
Wavelength (nm)	0.099
Sample environment	Quartz glass capillary, 1 mm ø
Absolute scaling method	Comparison with scattering from pure H_2O
Normalization	To transmitted intensity by beam-stop counter
Scattering intensity scale	Absolute scale, cm^{-1}
s range (nm^{-1}), ($s = 4\pi\sin(\theta)/\lambda$)	0.025–5.5
**Sample**	**1-Deoxy-D-xylulose-5-phosphate reductoisomerase (*Tg*DXR)**
Organism	*Toxoplasma gondii* (ME49)
UniProt ID	*V5B5Y5*
Mode of measurement	SEC-SAXS
SEC-Column	Superdex 200 increase 10/300
Flowrate (ml/min)	0.5
Injection volume (µl)	300
Temperature (°C)	10
Exposure time (# frames)	2 s (1500 frames)
# frames used for averaging	35
Protein buffer	20 mM Tris/HCl, 150 mM NaCl, 40 mM $MgCl_2$, 2% glycerol, pH 7.5
Protein concentration (mg/ml)	8.00
**Structural parameters**	
Guinier Analysis (PRIMUS)	
$I(0) \pm \sigma$ (cm^{-1})	54.11 ± 0.033
$R_g \pm \sigma$ (nm)	3.33 ± 0.0032
s-range (nm^{-1})	0.140 – 0.387
min < sRg < max limit	0.47 – 1.29
Data point range	1 - 49
Linear fit assessment (R^2)	0.9996
PDDF/P(r) Analysis (GNOM 5)	
$I(0) \pm \sigma$ (cm^{-1})	53.99 ± 0.032
$R_g \pm \sigma$ (nm)	3.31 ± 0.0025

D_{max} (nm)	10.44
Porod volume (nm^3)	176.01
s-range (nm^{-1})	0.140 – 5.029
$\chi 2$ / CorMap P-value	1.207 / 0.108
**Molecular mass (kDa)**	
From *I*(0)	not determined
From Qp (11)	105.53
From MoW2 (12)	108.24
From Vc (13)	101.69
Bayesian Inference (14)	104.90
From sequence	51.81 (monomer), 103.62 (dimer)
**Modelling**	
**EOM (Ensemble Optimisation Method)**	
Constant subtraction	0.007
s-range for fit (s_{min} – s_{max}; nm^{-1})	0.140 – 4.988
No. of representative structures	3
χ^2, CorMap *P*-value	1.259 / 0.108
**SASBDB accession codes**	SASDS47
**Software**	
ATSAS Software Version (15)	3.0.5
Primary data reduction	CHROMIXS (16)/ PRIMUS (17)
Data processing	GNOM (18)
Ensemble modelling	EOM (19, 20)
Model visualization	PyMOL (21)

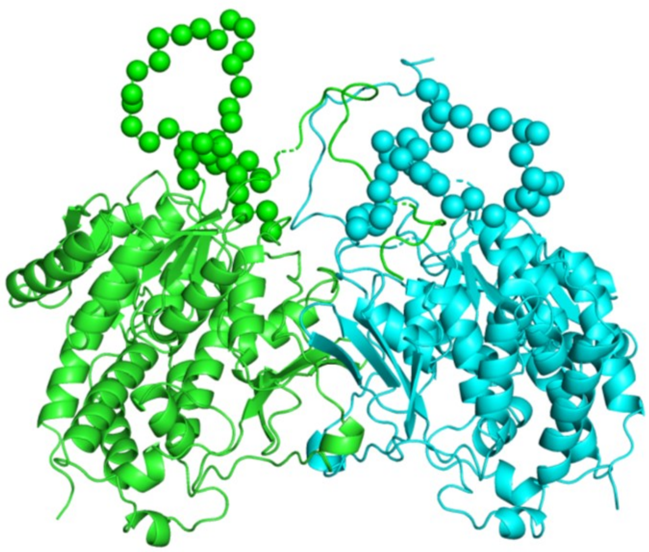

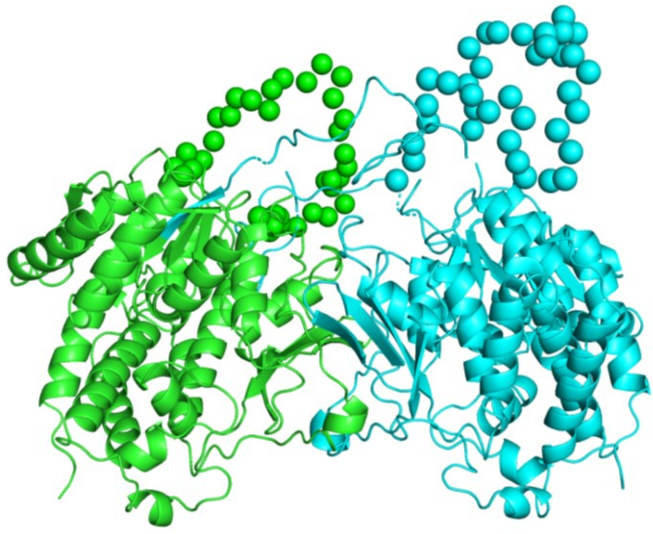

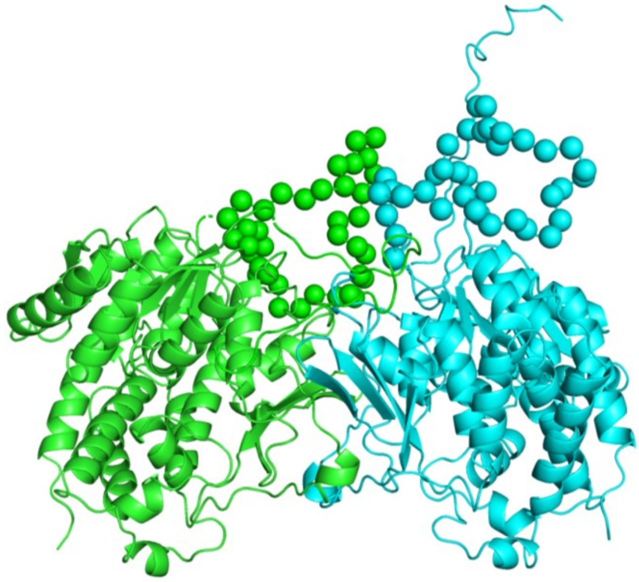

Supplementary Figure S4. Overlay view from the three EOM calculated models.

The rigid body protomers of *Tg*DXR from the crystal are shown in green and cyan cartoon representation. The loop region of each protomer is shown in spheres representation. The upper model corresponds to a volume fraction of 12 %, the middle on to a volume fraction of 25 % and the lower one to a volume fraction of 62 %.

5 Biological Data

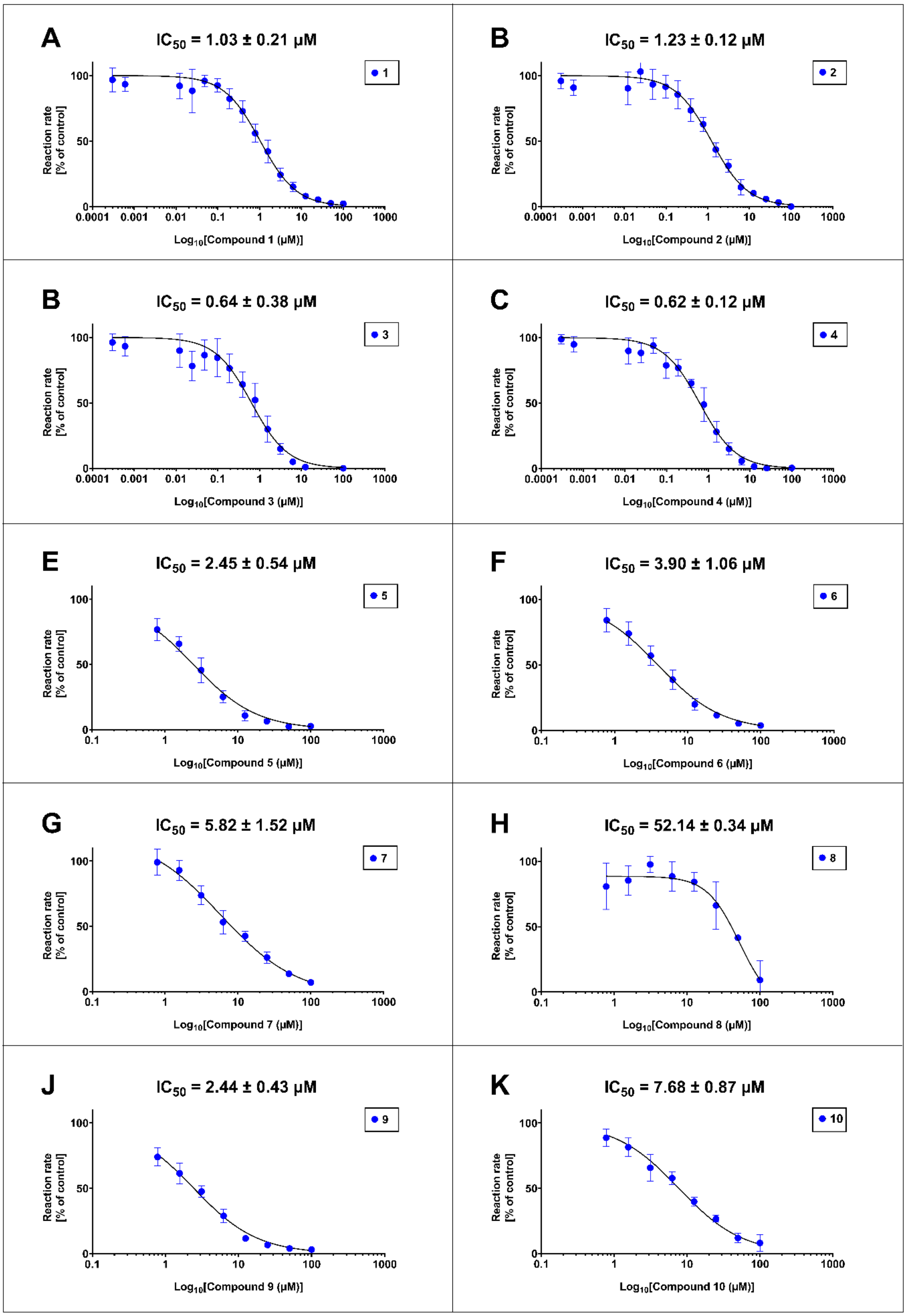

Supplementary Figure S5. ***In vitro*** **enzymatic inhibition of** ***Tg*****DXR of investigated compounds.**

The enzymatic inhibitory activity of **1** (**A**), **2** (**B**), **3** (**C**), **4** (**D**), **5** (**E**), **6** (**F**), **7** (**G**), **8** (**H**), **9** (**J**) and **10** (**K**) were determined by enzymatic assays *in vitro*. Experiments were conducted in 96 well plates at 30 °C containing 100 nM of purified *Tg*DXR protein in dimeric state, 100 µM of NADPH and 4 mM of $MgCl_2$ as cofactors, 100 µM of DXP as substrate in 50 mM HEPES buffer (pH 7.5) containing 50 µg/mL of bovine serum albumin (BSA). The investigated compounds were tested in serial dilution 1:2. Data shown are from the means of three independent experiments each performed in duplicate ($n = 6$) ± S.D. IC_{50} of each compound are shown.

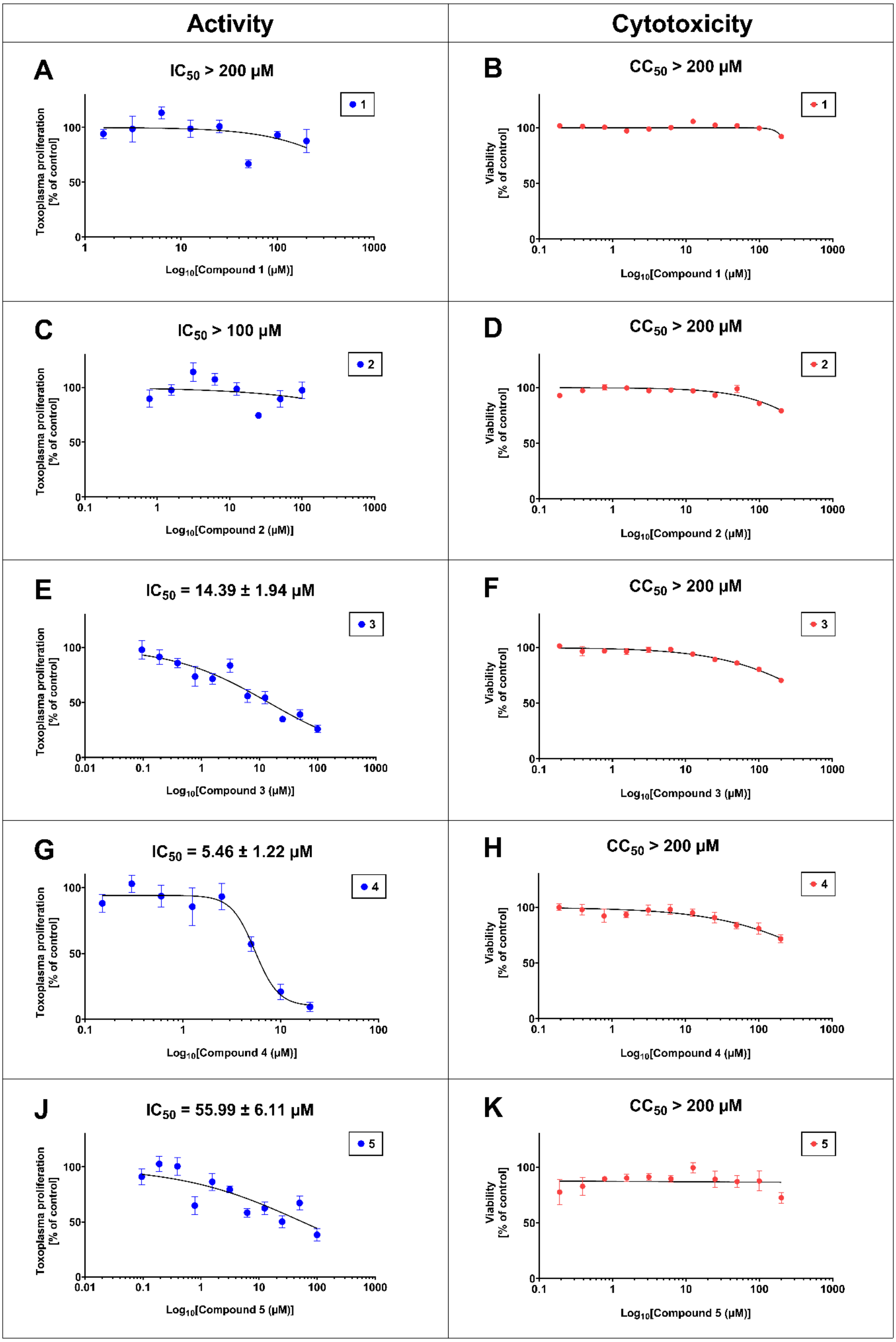
Activity
Cytotoxicity
A
IC50 > 200 µM
Toxoplasma proliferation [% of control]
Log10[Compound 1 (µM)]
1
B
CC50 > 200 µM
Viability [% of control]
Log10[Compound 1 (µM)]
1
C
IC50 > 100 µM
Toxoplasma proliferation [% of control]
Log10[Compound 2 (µM)]
2
D
CC50 > 200 µM
Viability [% of control]
Log10[Compound 2 (µM)]
2
E
IC50 = 14.39 ± 1.94 µM
Toxoplasma proliferation [% of control]
Log10[Compound 3 (µM)]
3
F
CC50 > 200 µM
Viability [% of control]
Log10[Compound 3 (µM)]
3
G
IC50 = 5.46 ± 1.22 µM
Toxoplasma proliferation [% of control]
Log10[Compound 4 (µM)]
4
H
CC50 > 200 µM
Viability [% of control]
Log10[Compound 4 (µM)]
4
J
IC50 = 55.99 ± 6.11 µM
Toxoplasma proliferation [% of control]
Log10[Compound 5 (µM)]
5
K
CC50 > 200 µM
Viability [% of control]
Log10[Compound 5 (µM)]
5

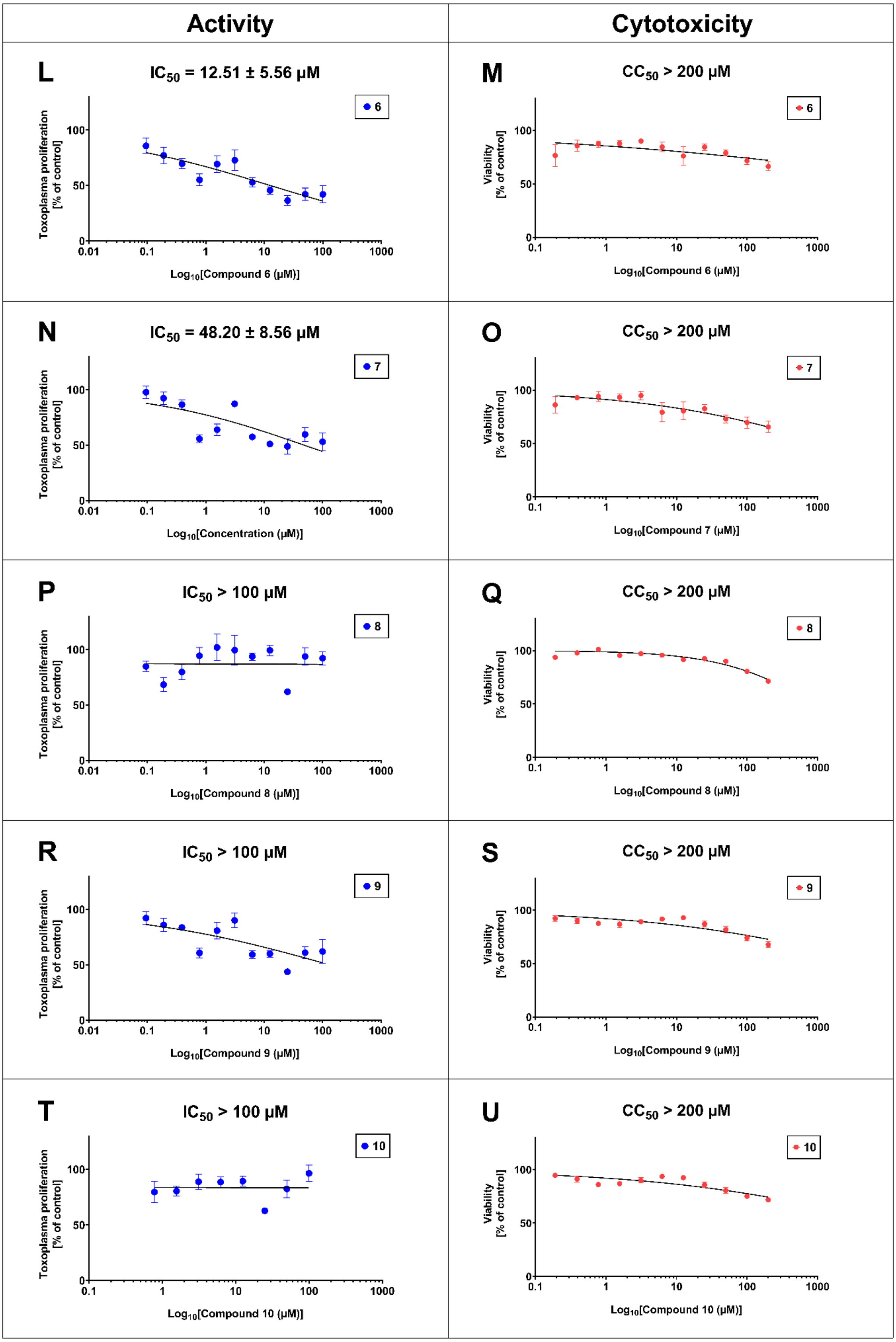
Activity
Cytotoxicity
L
IC50 = 12.51 ± 5.56 µM
6
Toxoplasma proliferation [% of control]
Log10[Compound 6 (µM)]
M
CC50 > 200 µM
6
Viability [% of control]
Log10[Compound 6 (µM)]
N
IC50 = 48.20 ± 8.56 µM
7
Toxoplasma proliferation [% of control]
Log10[Concentration (µM)]
O
CC50 > 200 µM
7
Viability [% of control]
Log10[Compound 7 (µM)]
P
IC50 > 100 µM
8
Toxoplasma proliferation [% of control]
Log10[Compound 8 (µM)]
Q
CC50 > 200 µM
8
Viability [% of control]
Log10[Compound 8 (µM)]
R
IC50 > 100 µM
9
Toxoplasma proliferation [% of control]
Log10[Compound 9 (µM)]
S
CC50 > 200 µM
9
Viability [% of control]
Log10[Compound 9 (µM)]
T
IC50 > 100 µM
10
Toxoplasma proliferation [% of control]
Log10[Compound 10 (µM)]
U
CC50 > 200 µM
10
Viability [% of control]
Log10[Compound 10 (µM)]

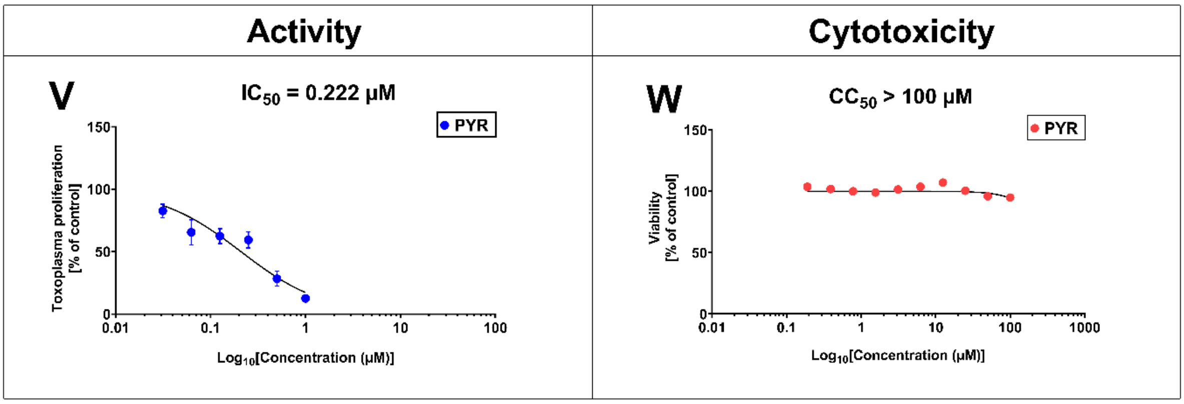

Supplementary Figure S6. Anti-toxoplasma activity and cytotoxicity on human Hs27 fibroblasts of the investigated compounds.

The inhibitory activities of **1** (**A**), **2** (**C**), **3** (**E**), **4** (**G**), **5** (**J**), **6** (**L**), **7** (**N**), **8** (**P**), **9** (**R**), **10** (**T**) and **PYR** (**V**) were determined by the *T. gondii in vitro* inhibition assay via the [^{3}H]-uracil incorporation into the RNA of the parasite. Cytotoxicity of **1** (**B**), **2** (**D**), **3** (**F**), **4** (**H**), **5** (**K**), **6** (**M**), **7** (**O**), **8** (**Q**), **9** (**S**), **10** (**U**) and **PYR** (**W**) were measured by MTT assays on human Hs27 fibroblasts. Data shown are from the means of three independent experiments each performed in duplicate (n = 6) ± SEM. IC_{50} ± S.D. and CC_{50} values of each compound are shown.

6 References

1. Kunfermann A, Lienau C, Illarionov B, Held J, Gräwert T, Behrendt CT, et al. IspC as target for antiinfective drug discovery: synthesis, enantiomeric separation, and structural biology of fosmidomycin thia isosters. Journal of medicinal chemistry. 2013;56(20):8151-62. DOI: 10.1021/jm4012559

2. Lienau C, Gräwert T, Avelar LAA, Illarionov B, Held J, Knaab TC, et al. Novel reverse thia-analogs of fosmidomycin: Synthesis and antiplasmodial activity. European Journal of Medicinal Chemistry. 2019;181:111555. DOI: 10.1016/j.ejmech.2019.07.058.

3. Brücher K, Illarionov B, Held J, Tschan S, Kunfermann A, Pein MK, et al. α-Substituted β-Oxa Isosteres of Fosmidomycin: Synthesis and Biological Evaluation. Journal of Medicinal Chemistry. 2012;55(14):6566-75. DOI: 10.1021/jm300652f.

4. Mac Sweeney A, Lange R, Fernandes RP, Schulz H, Dale GE, Douangamath A, et al. The crystal structure of E.coli 1-deoxy-D-xylulose-5-phosphate reductoisomerase in a ternary complex with the antimalarial compound fosmidomycin and NADPH reveals a tight-binding closed enzyme conformation. J Mol Biol. 2005;345(1):115-27. DOI: 10.1016/j.jmb.2004.10.030.

5. Andaloussi M, Henriksson LM, Więckowska A, Lindh M, Björkelid C, Larsson AM, et al. Design, Synthesis, and X-ray Crystallographic Studies of α-Aryl Substituted Fosmidomycin Analogues as Inhibitors of Mycobacterium tuberculosis 1-Deoxy-d-xylulose 5-Phosphate Reductoisomerase. Journal of Medicinal Chemistry. 2011;54(14):4964-76. DOI: 10.1021/jm2000085.

6. Sooriyaarachchi S, Chofor R, Risseeuw MD, Bergfors T, Pouyez J, Dowd CS, et al. Targeting an Aromatic Hotspot in Plasmodium falciparum 1-Deoxy-d-xylulose-5-phosphate Reductoisomerase with β-Arylpropyl Analogues of Fosmidomycin. ChemMedChem. 2016;11(18):2024-36. DOI: 10.1002/cmdc.201600249.

7. Cai G, Deng L, Xue J, Moreno SN, Striepen B, Song Y. Expression, characterization and inhibition of Toxoplasma gondii 1-deoxy-D-xylulose-5-phosphate reductoisomerase. Bioorganic & medicinal chemistry letters. 2013;23(7):2158-61. DOI: 10.1016/j.bmcl.2013.01.097.

8. Jomaa H, Wiesner J, Sanderbrand S, Altincicek B, Weidemeyer C, Hintz M, et al. Inhibitors of the nonmevalonate pathway of isoprenoid biosynthesis as antimalarial drugs. Science. 1999;285(5433):1573-6. DOI: 10.1126/science.285.5433.1573.

9. Pernot P, Theveneau P, Giraud T, Fernandes RN, Nurizzo D, Spruce D, et al. New beamline dedicated to solution scattering from biological macromolecules at the ESRF. Journal of Physics: Conference Series. 2010;247(1):012009. DOI: 10.1088/1742-6596/247/1/012009.

10. Pernot P, Round A, Barrett R, De Maria Antolinos A, Gobbo A, Gordon E, et al. Upgraded ESRF BM29 beamline for SAXS on macromolecules in solution. Journal of synchrotron radiation. 2013;20(Pt 4):660-4. DOI: 10.1107/S0909049513010431.

11. Porod G. Die Röntgenkleinwinkelstreuung Von Dichtgepackten Kolloiden Systemen - 1 Teil. Kolloid Z Z Polym. 1951;124(2):83-114. DOI: 10.1007/Bf01512792.

12. Fischer H, Neto MD, Napolitano HB, Polikarpov I, Craievich AF. Determination of the molecular weight of proteins in solution from a single small-angle X-ray scattering measurement on a relative scale. Journal of Applied Crystallography. 2010;43:101-9. DOI: 10.1107/S0021889809043076.

13. Rambo RP, Tainer JA. Accurate assessment of mass, models and resolution by small-angle scattering. Nature. 2013;496(7446):477-81. DOI: 10.1038/nature12070.

14. Hajizadeh NR, Franke D, Jeffries CM, Svergun DI. Consensus Bayesian assessment of protein molecular mass from solution X-ray scattering data. Sci Rep. 2018;8(1):7204. DOI: 10.1038/s41598-018-25355-2.

15. Manalastas-Cantos K, Konarev PV, Hajizadeh NR, Kikhney AG, Petoukhov MV, Molodenskiy DS, et al. ATSAS 3.0: expanded functionality and new tools for small-angle scattering data analysis. Journal of Applied Crystallography. 2021;54(1). DOI: 10.1107/S1600576720013412.

16. Panjkovich A, Svergun DI. CHROMIXS: automatic and interactive analysis of chromatography-coupled small angle X-ray scattering data. Bioinformatics. 2017. DOI: 10.1093/bioinformatics/btx846.

17. Konarev PV, Volkov VV, Sokolova AV, Koch MHJ, Svergun DI. PRIMUS: a Windows PC-based system for small-angle scattering data analysis. Journal of Applied Crystallography. 2003;36:1277-82. DOI: 10.1107/S0021889803012779.

18. Svergun DI. Determination of the Regularization Parameter in Indirect-Transform Methods Using Perceptual Criteria. Journal of Applied Crystallography. 1992;25:495-503. DOI: 10.1107/S0021889892001663.

19. Tria G, Mertens HD, Kachala M, Svergun DI. Advanced ensemble modelling of flexible macromolecules using X-ray solution scattering. IUCrJ. 2015;2(Pt 2):207-17. DOI: 10.1107/S205225251500202X.

20. Bernado P, Mylonas E, Petoukhov MV, Blackledge M, Svergun DI. Structural characterization of flexible proteins using small-angle X-ray scattering. J Am Chem Soc. 2007;129(17):5656-64. DOI: 10.1021/ja069124n.

21. PyMOL. The PyMOL Molecular Graphics System, Version 2.5 Schrödinger, LLC. 2022.
